# Metabolic Profile, Bioactivities, and Variations in the Chemical Constituents of Essential Oils of the *Ferula* Genus (Apiaceae)

**DOI:** 10.3389/fphar.2020.608649

**Published:** 2021-03-12

**Authors:** Priyankaraj Sonigra, Mukesh Meena

**Affiliations:** Laboratory of Phytopathology and Microbial Biotechnology, Department of Botany, Mohanlal Sukhadia University, Udaipur, India

**Keywords:** antimicrobial, antioxidant, cytotoxic, essential oils, *Ferula* species, phytochemicals

## Abstract

The genus *Ferula* is the third largest and a well-known genus of the Apiaceae family. It is categorized in the Peucedaneae tribe and Ferulinae subtribe of the Apiaceae family. At present, about 180 *Ferula* species have been reported. The genus is mainly distributed throughout central and South-West Asia (especially Iran and Afghanistan), the far-East, North India, and the Mediterranean. The genus *Ferula* is characterized by the presence of oleo-gum-resins (asafoetida, sagapenum, galbanum, and ammoniacum) and their use in natural and conventional pharmaceuticals. The main phytochemicals present in the genus *Ferula* are as follows: coumarin, coumarin esters, sesquiterpenes, sesquiterpene lactones, monoterpene, monoterpene coumarins, prenylated coumarins, sulfur-containing compounds, phytoestrogen, flavonoids and carbohydrates. This genus is considered to be a valuable group of medicinal plants due to its many different biological and pharmacological uses as volatile oils (essential oils). Numerous biological activities are shown by the chemical components of the essential oils obtained from different *Ferula* species. Because this genus includes many bioactivities such as antimicrobial, insecticidal, antioxidant, cytotoxic, etc., researchers are now focusing on this genus. Several reviews are already available on this particular genus, including information about the importance and the uses of all the phytochemicals found in the species of *Ferula*. Despite this, no review that specifically provides information about the biological activities of *Ferula*-derived essential oils, has been published yet. Therefore, the present review has been conducted to provide important information about the chemical profile, factors affecting the chemical composition, and biological activities of essential oils of the *Ferula* species.

## Introduction

Medicinal plants are considered to be an invaluable and a constant source of biologically active phytochemicals. Amid all the other medicinal plants, spices are an important element of Ayurveda. Instead of being an irreplaceable part of food accessories, they have their history in the field of folk medicine since they possess different phytochemicals (Amalraj and Gopi, 2017). These phytochemicals have long been used to cure several ailments, and they can be a promising alternative to conventional medical therapies (Upadhyay, 2017). The Apiaceae family consists of about 455 genera and 3,600–3,751 plants species that belong to this family are often used in the form of spices. *Ferula* is the 3rd largest genus of the Apiaceae family and is categorized in the Peucedaneae tribe and Ferulinae subtribe of the family. At present, about 180 *Ferula* species have been reported. *Ferula* is a Latin word meaning “vehicle” or “carrier” (Iranshahy and Iranshahi, 2011). The genus has a wide distribution all over central and South–West Asia (especially Iran and Afghanistan), the far-East, North India, and the Mediterranean (Hosseinzadeh et al., 2020a; Hosseinzadeh et al., 2020b), and some are distributed in desert areas. Most of the *Ferula* species grow in mountainous regions and arid climates (Yaqoob and Nawchoo, 2016). The *Ferula* species has been of great importance in folk and traditional medicine for more than a 1,000 years. The genus *Ferula* is characterized by the presence of oleo-gum-resins (asafoetida, sagapenum, galbanum, and ammoniacum) (Ahmadi et al., 2020).

The main chemical constituents present in the genus *Ferula* are as follows: coumarin (ferulenol, galbanic acid and umbelliprenin), coumarin esters (ferulone A, B), sesquiterpenes (germacranes, himachalanes, carotanes, humulanes, guaianes, daucane esters farnesiferol A and B, and sinkiangenorin C and E) (Zellagui et al., 2012), sesquiterpene lactones, monoterpene (*α*-pinene, *β*-pinene), monoterpene coumarins (auraptene), prenylated coumarins (ferprenin), sulfur-containing derivatives, phytoestrogen (ferutinin), flavonoids, carbohydrates (galactose, glucuronic acid, arabinose, rhamnose) (Iranshahi et al., 2018; Mohammadhosseini et al., 2019a; Salehi et al., 2019). Generally, aromatic acid lactones sesquiterpenes, coumarins, and sesquiterpene coumarins are present in the roots of *Ferula* species (Teng et al., 2013), whereas monoterpenes, oxygenated monoterpenoids sesquiterpenes and oxygenated sesquiterpenoid are the main chemical constituents of essential oil present in the aerial parts of *Ferula* (Mohammadhosseini et al., 2015). Due to the widespread therapeutic effects of this genus, it is being used in folk medicine to treat a variety of diseases and disorders, including skin infections, psychiatric disorders (especially seizure), hyperlipidemia, diabetes, arteries sclerosis, digestive disorders (dysentery), osteoporosis, arthritis, HIV, influenza type A, cancers (uterine cancer), muscle relaxant, rheumatism, headaches, hypertension, toothache and dizziness (Zhou et al., 2017; Meena et al., 2018; Arjmand and Dastan, 2020; Esmaeili et al., 2020).

In the last few decades, research on the phytochemicals of the *Ferula* species have gained momentum due to their natural origin, effectiveness and low to no unpleasant side effects. In recent years, several biological activities of phytochemicals, isolated from various *Ferula* species found in different geographical regions, have been reported, such as antimicrobial (Utegenova et al., 2018; Kahraman et al., 2019), insecticidal (Liu et al., 2020; Pavela et al., 2020), aphicidal (Stepanycheva et al., 2012), antihelmintic (Tavassoli et al., 2018), antiprotozoal activity (Bashir et al., 2014; Amin et al., 2016), antimycobacterial (Fallah et al., 2015), antiviral (Zhai et al., 2012; Ghannadi et al., 2014), antioxidant (Deveci et al., 2018; Rahali et al., 2019), anticancer (Asemani et al., 2018; Hosseinzadeh et al., 2020a; Hosseinzadeh et al., 2020b), antitumor (Alizadeh et al., 2018), cytotoxic (Iranshahy et al., 2019; Mahaki et al., 2019), antiproliferative (Verma et al., 2019), acetylcholinesterase inhibitory (Deveci et al., 2018; Karakaya et al., 2019a), antidepressant (Mohammadhosseini, 2016), antiulcer (Bagheri et al., 2018), muscarinic receptors inhibitory (Khazdair et al., 2015; Gholamnezhad et al., 2018), antihypertensive (Safaeian et al., 2015), anti-epileptic (Kiasalari et al., 2013), antispasmodic (Pavlović et al., 2012), antinociceptive (Bagheri et al., 2014a), phytotoxic (Dastan et al., 2014), hypnotic (Abbasnia and Aeinfar, 2016), antihemolytic and antioxidant (Nabavi et al., 2011), anticoagulant (Han et al., 2010), anticonvulsant (Bagheri at al., 2010; Bagheri et al., 2014a; Bagheri et al., 2014b; Bagheri et al., 2014c) relaxant (Bayrami et al., 2013; Bagheri et al., 2014a; Bagheri et al., 2014b; Bagheri et al., 2014c), memory enhancement (Upadhyay, 2017), increasing digestive enzyme activity (Safari et al., 2019), antigenotoxic (Ozkan et al., 2014; Rezaee et al., 2014), antihyperlipidemic (Yusufoglu et al., 2015; Latifi et al., 2019), antihyperglycemic (Iranshahi and Alizadeh, 2012; Yusufoglu et al., 2015), antidiabetic (Yarizade et al., 2017; Latifi et al., 2019), anxiolytics (Upadhyay et al., 2014; Batra et al., 2020) and antihepatotoxicity (Fatima et al., 2017; Deniz et al., 2019). In these activities, essential oils also make up a minimal but significant share.

Essential oils are defined as volatile aromatic compounds that provide a distinctive flavor, aroma, or scent to a plant (Raut and Karuppayil, 2014; Meena et al., 2017a; Chandran et al., 2020a, Chandran et al., 2020b). They are hydrophobic, lipid-soluble liquid comprised of volatile and non-volatile fractions (Hussain et al., 2008). The volatile fraction includes mono and sesquiterpene components, their oxygenated derivatives, alcohols, aliphatic aldehydes, and esters. On the other hand, non-volatile residues contain carotenoids, flavonoids, fatty acids, and waxes (Aziz et al., 2018). Essential oils are the by-products of plants’ secondary metabolic processes. Essential oils can be synthesized by any of the plant organs such as leaves, buds, flowers stems, fruits, seeds, wood or bark and roots, and are stored in epidermis cells, secretory cells, glandular hairs and plant-cell wall in the form of small droplets (Prakash et al., 2015; Barupal et al., 2019; Mohammadhosseini et al., 2019b; Chandran et al., 2020a). Generally, *Ferula* species found in warm to temperate climates such as tropical and Mediterranean countries possess essential oils. *Ferula* deputize an important part of the conventional pharmacopeia of those countries (Bakkali et al., 2008; Meena and Samal, 2019). In this review, we intend to cover different biological activities and clinical applications of essential oils of the *Ferula* genus described in recent years. Although, many reviews are available on the medicinal and bioactivity of the *Ferula* species, no review which specifically provides detailed and encompassing information about the biological activities and pharmaceutical applications of various essential oils has been published yet.

## Phytochemistry of Essential Oils and Factors Affecting the Chemical Constituents of the Essential Oils

**FIGURE 1:**
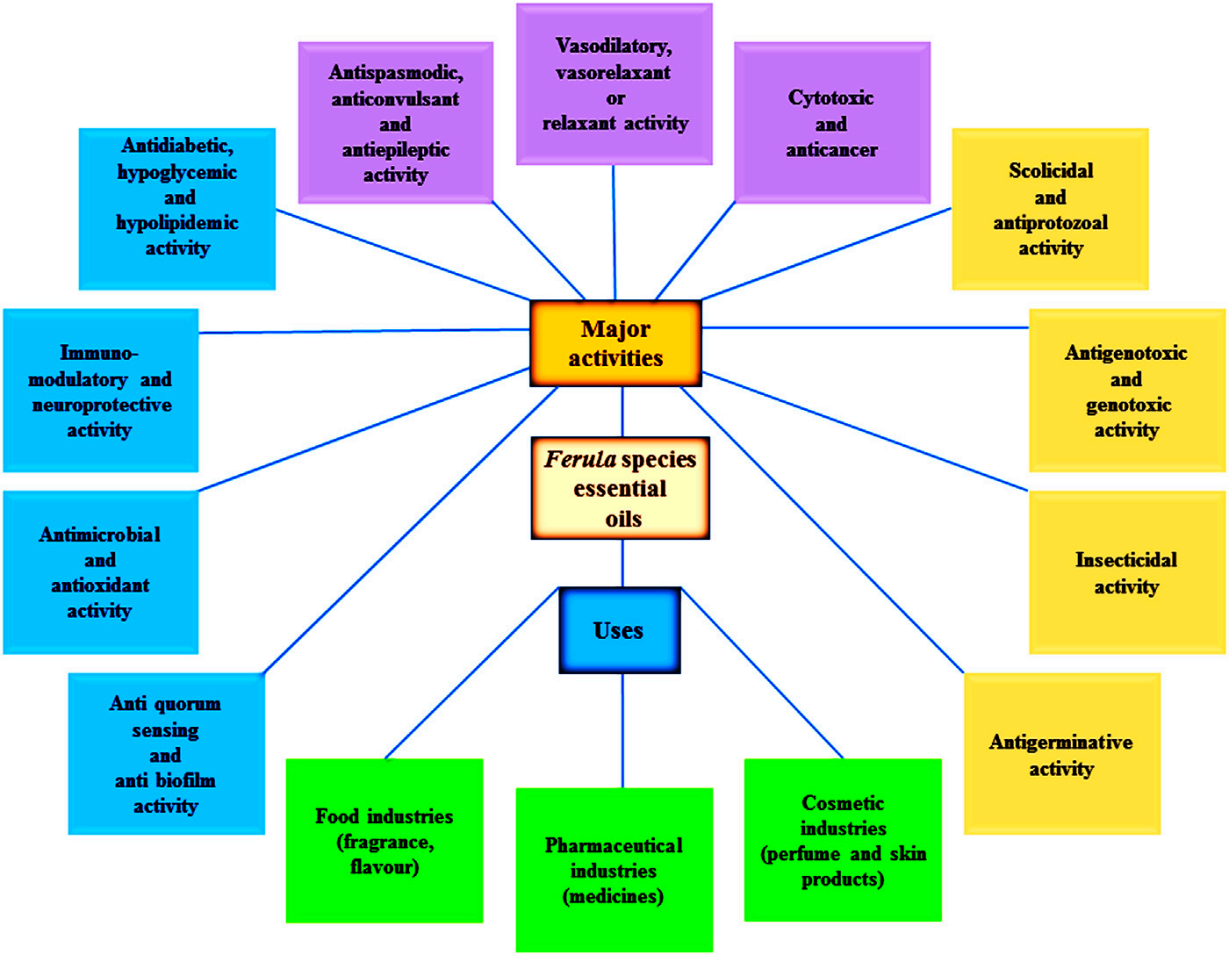
Different bioactivities and uses of the *Ferula* species essential oils.

**FIGURE 2:**
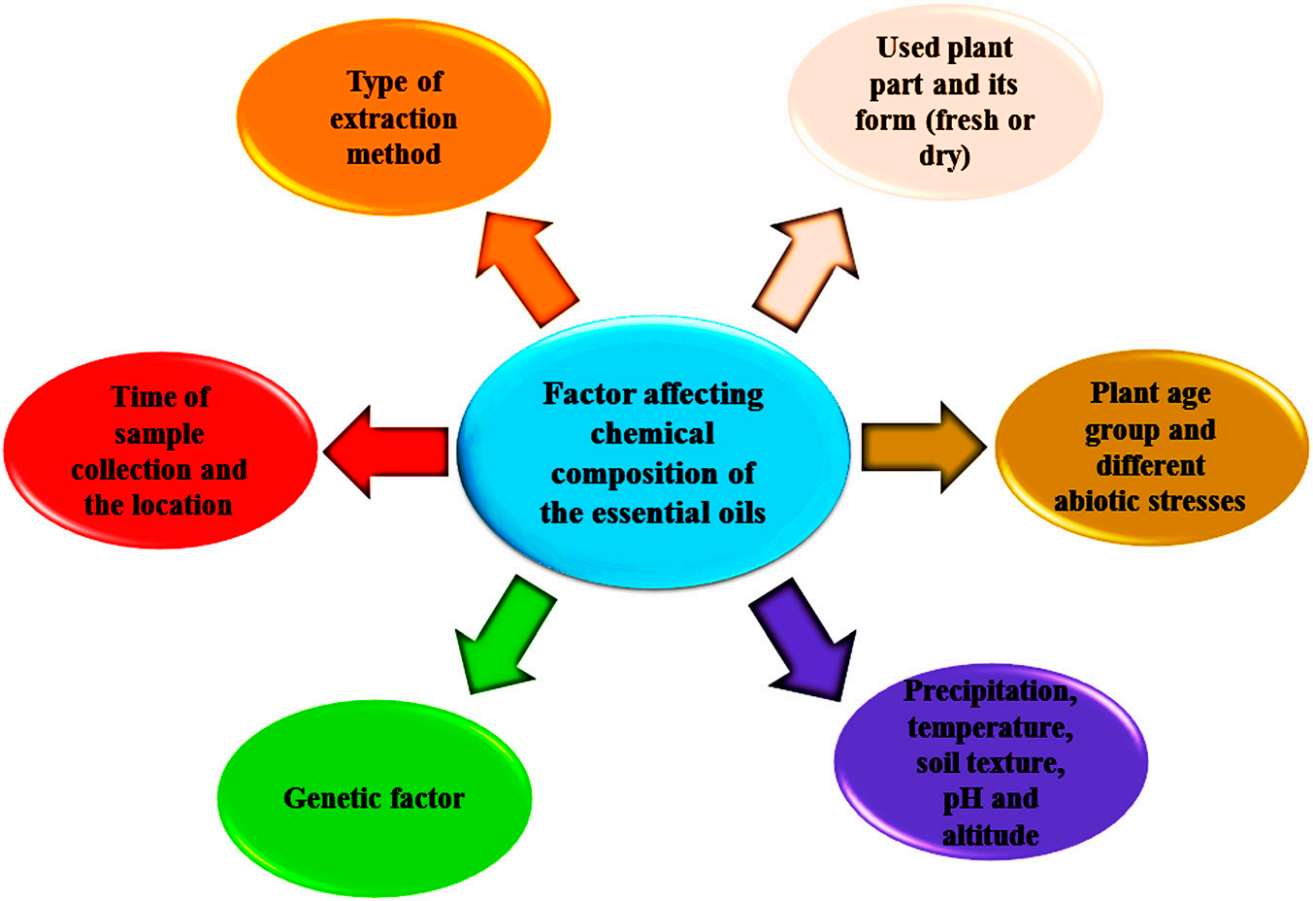
Factors affecting the chemical profile of essential oils of *Ferula* species.

Essential oils can be considered as liquids that are lighter than water and represent remarkable hydrophobic characteristics involving a vast number of valuable natural compounds (Mohammadhosseini et al., 2019b). These secondary metabolites can be extracted from different parts of the plant materials using a variety of classical and advanced methods (Mohammadhosseini, 2017). Approximately 160 chemical compounds have been identified in the essential oils of the *Ferula* species; these chemical compounds are responsible for all biological activities represented by the essential oils and make them a better choice for industrial purposes (Figure 1). These chemical groups are monoterpene hydrocarbons: limonene, myrcene, *γ*-terpinene, *p*-cymene, *δ*-3-carene, *α*-pinene, and *β*-pinene (Znati et al., 2012; Ben et al., 2016; Khalifaev et al., 2018; Malekzadeh et al., 2018; Asilbekova et al., 2019; Karakaya et al., 2019b; Meena and Swapnil, 2019; Tabari et al., 2019, Topdas et al., 2020); oxygenated monoterpenoids: *α*-terpinyl acetate, linalool, sabinene, *α*-terpineol, verbenone, neryl acetate and ar-curcumene (Znati et al., 2012; Radulović et al., 2013; Essid et al., 2015; Khoury et al., 2018), sesquiterpene hydrocarbons: germacrene B and D, *β*-caryophyllene (E)-caryophyllene, bicyclogermacrene,*α*-gurjunene,*γ*-elemene, *γ*-cadinene and *δ*-cadinene (Zellagui et al., 2012; Essid et al., 2015; Meena et al., 2017b; Topdas et al., 2020; Ahmadi et al., 2020); oxygenated sesquiterpenoids: *α*-cadinol, caryophyllene oxide, guaiol, *α*-eudesmol (Z)-ocimenone (E)-nerolidol (E)-ocimenone, viridiflorol, carotol, epi-*α*-muurolol, hinesol, valerianol and spathulenol (Benchabane et al., 2012; Kasaian et al., 2016; Deveci et al., 2018; Akhzari and Saadatfar, 2019; Baccari et al., 2020; Meena et al., 2020a, Meena et al., 2020b) and sulfur-containing metabolites: dimethyl-trisulphide (E)-1-propenylsec-butyl disulfide, sec-butyl-(Z)-propenyl-disulphide, di-sec-butyl-disulphide,phenol 2-methyl-5-(1-methylethyl), sec-butyl-(E)-propenyl-disulphide, 2,5-diethylthiophene, trimethylthiophene and 1-methylpropyl-(1E)-prop-1-en-1-yl-disulfide,bis-[(1-methylthio) propyl]-disulfide and 1-methylpropyl-(Z)-prop-1-en-1-yl-disulfide (Kavoosi et al., 2013; Kasaian et al., 2016; Oüzek et al., 2017; Hassanabadi et al., 2019) (Table 1). The chemical profile of the essential oil (aerial parts) of *Ferula orientalis* L. was evaluated by Karakaya et al., (2019a). Pinene (*α*: 75.9% and *β*: 3.4%) was reported as an abundant constituent of the essential oil. Azarnivand et al., (2011) reported the variation in the chemical profile and yield of the essential oil extracted from the dry and fresh aerial parts of *Ferula ovina* (Boiss.) Boiss. In accordance with the research results, the amount of oil in the fresh aerial parts was higher than the dried aerial plant parts; the percentage of essential oil in the dry and fresh part of *F. ovina* was observed as 0.4% and 0.25%, respectively. The chemical profile of the essential oil in fresh (limonene, *α*-pinene, *β*-myrcene, *cis*-*β*-ocimene, isosylvestrene, *β*-pinene) and dried aerial parts (*α*-pinene, spathulenol, germacrene D, *β*-caryophyllene, *α*-terpineol and caryophyllene oxide) of *F. ovina* were also varied. In a report, it was observed that when *F. ovina* is consumed in its fresh form, it is poisonous while it is safe in dry form. The reason behind this was the presence of a high percentage of *β*-myrcene and limonene in new parts (Azarnivand et al., 2011). The environmental condition, soil texture, altitude, temperature, and precipitation rate also affect the oil content in *Ferula* species (Figure 2). Moghaddam and Farhadi (2015) revealed the correlation between mean annual temperatures and altitude; moreover, the effect of altitude on the yield and composition of essential oil was also studied. The maximum accumulations of essential oil and the presence of a higher number of sulfur compounds in essential oil were found in *Ferula assa-foetida* L. The study of chemical profiles of nine samples revealed that (E)-propenyl sec-butyl disulfide (37–54%) was the abundant compound with (Z)-propenyl sec-butyl disulfide (12–23%) in the essential oils (Hassanabadi et al., 2019). A study showed that decreasing temperature and increasing altitude had a negative correlation with the content of essential oils. In a cluster analysis, it was also concluded that genetic factors have had a greater effect on the chemical constituents of essential oil of *F. assa-foetida* L. compared to the environmental factor. The quality, quantity, and chemical profile of essential oils also differed in accordance with the methods of extraction (Mohammadhosseini and Nekoei, 2014; Kasaian et al., 2016).

**TABLE 1:** Chemical structures of some monoterpene hydrocarbons, oxygenated monoterpenoids, sesquiterpenes hydrocarbons, oxygenated sesquiterpenoids, sulfur containing compounds present in the essential of *Ferula* genus.

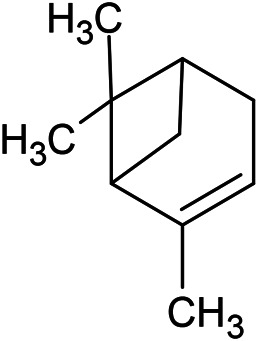	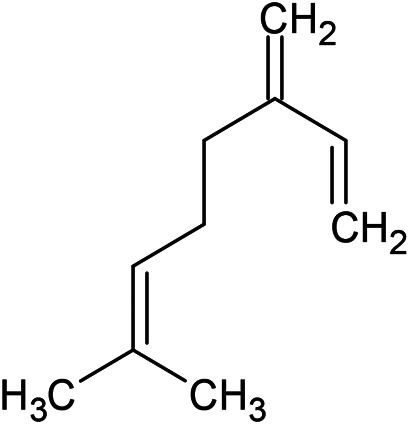					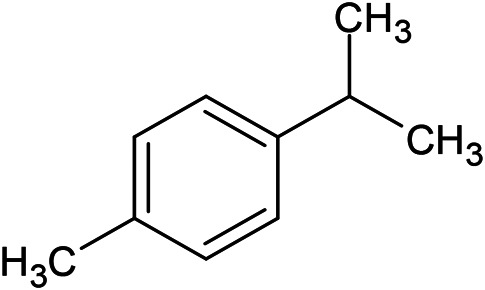					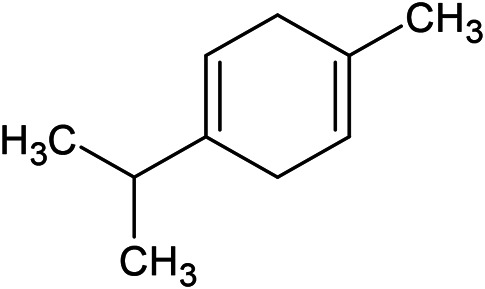		
*α*-Pinene	Myrcene					*p*-Cymene					δ-Terpinene		
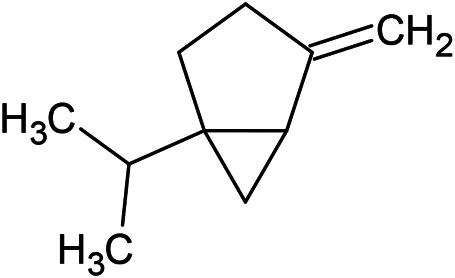	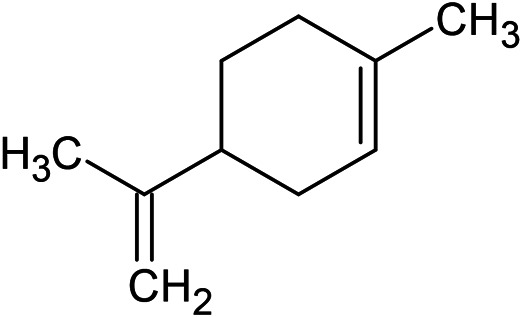					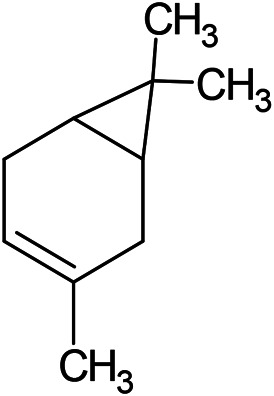							
Sabinene	Limonene					δ-3-Carene							
**Monoterpene hydrocarbons**													
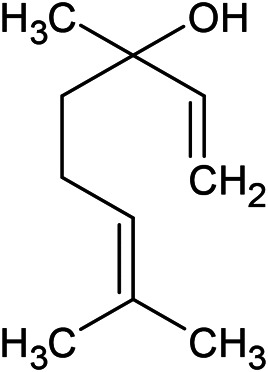		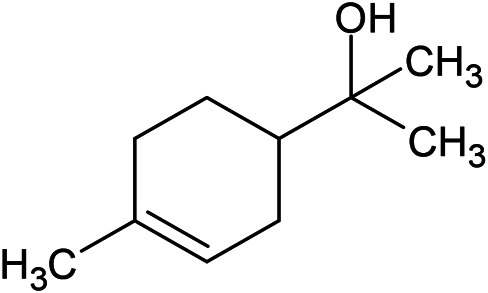			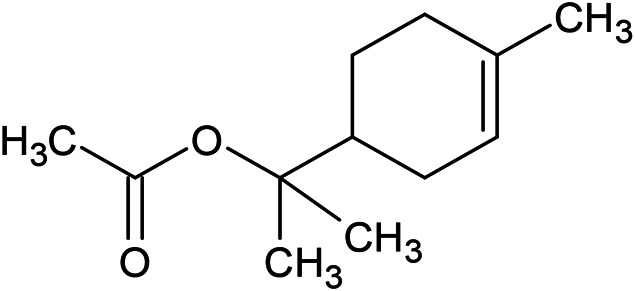						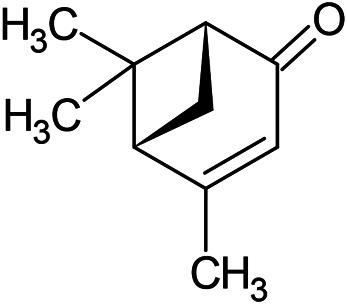		
Linalool		α-Terpineol			α-Terpinyl acetate						Verbenone		
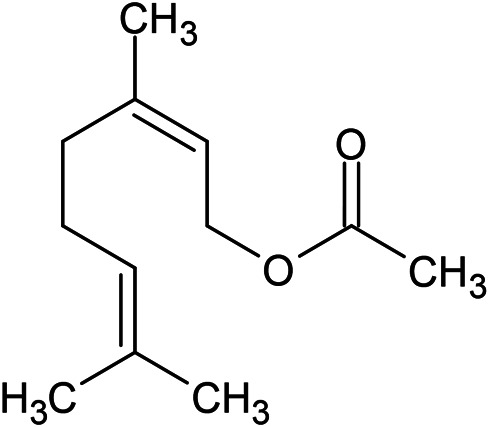													
Neryl acetate													
**Oxygenated monoterpenoids**													
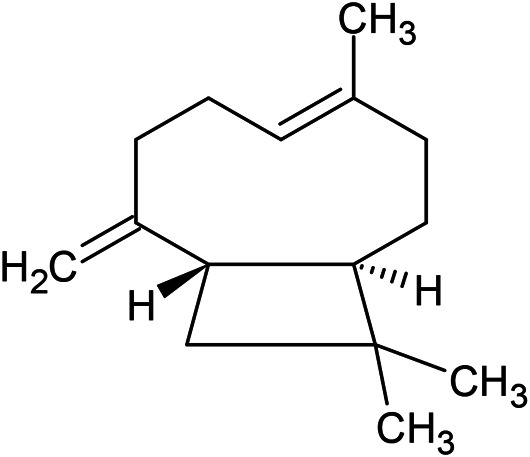		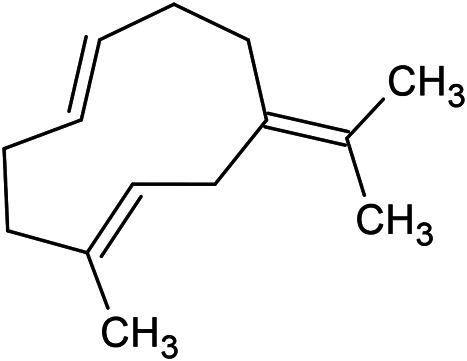					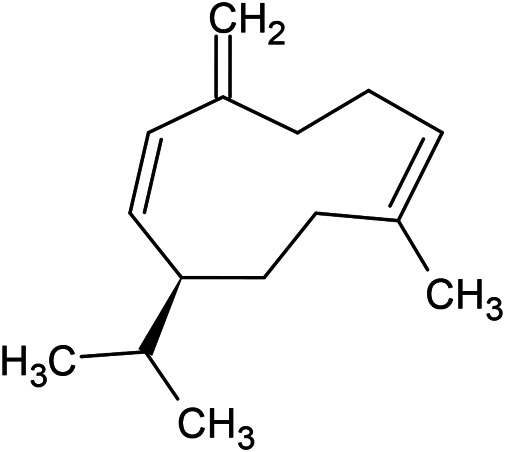			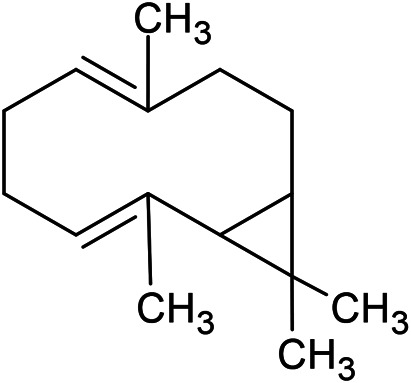			
β-Caryophyllene		Germacrene B					Germacrene D			Bicyclogermacrene			
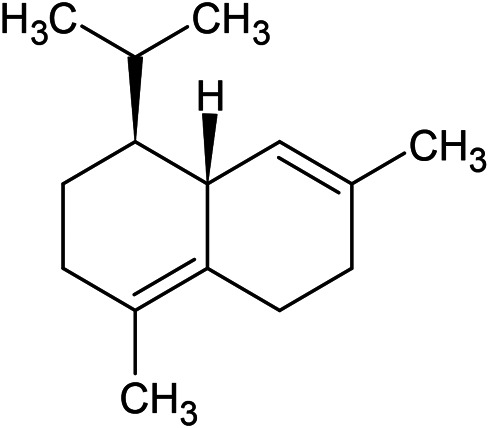		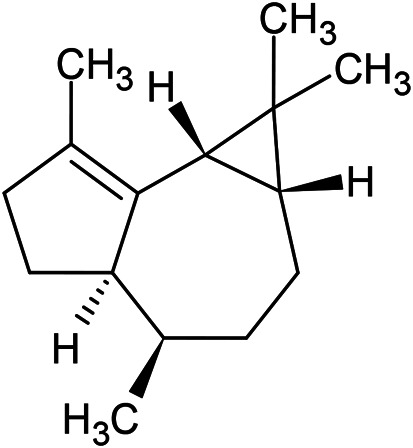					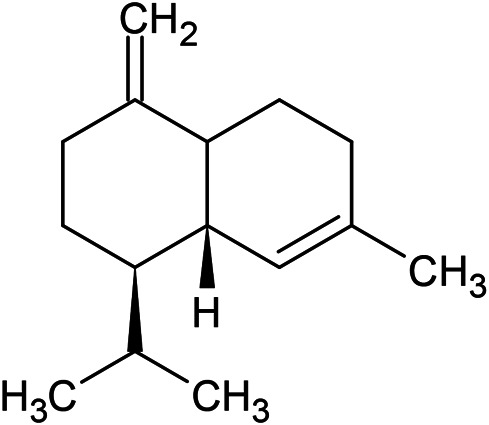			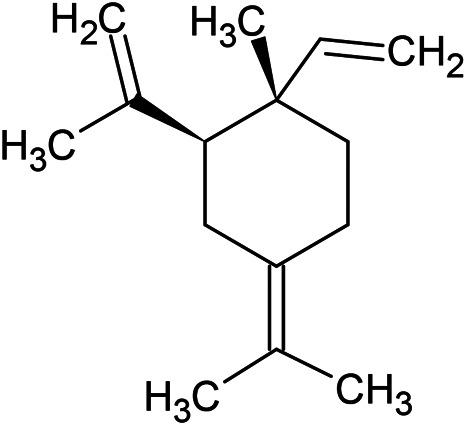			
δ-Cadinene		α-Gurjunene					γ-Cadinene			γ-Elemene			
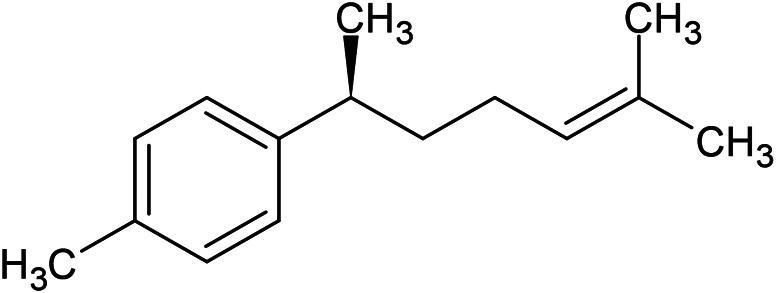													
(s)-Ar-curcumene													
**Sesquiterpene hydrocarbons**													
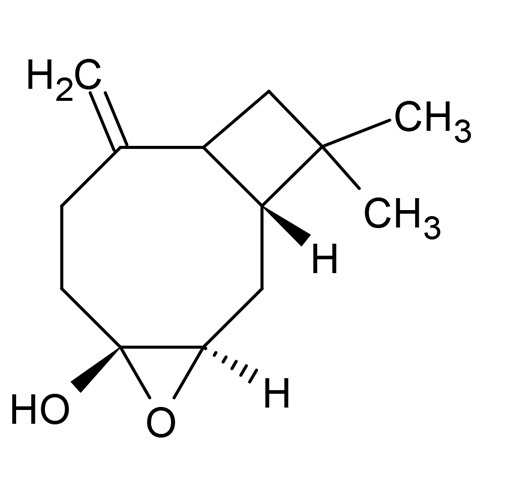			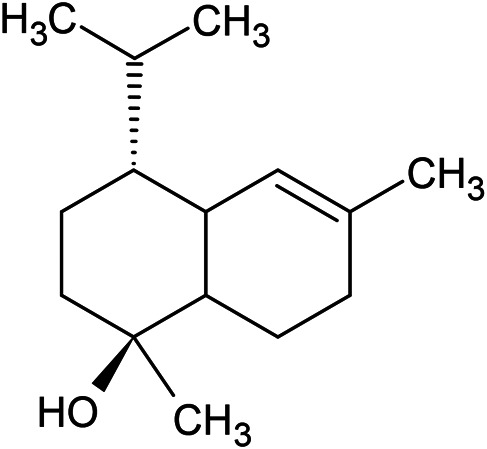						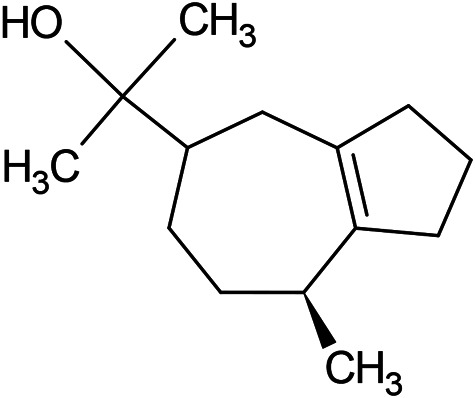				
Caryophyllene oxide			α-Cadinol						Guaiol				
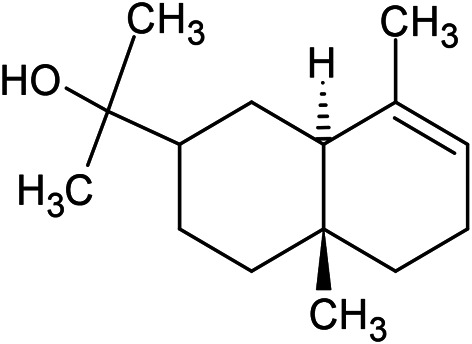			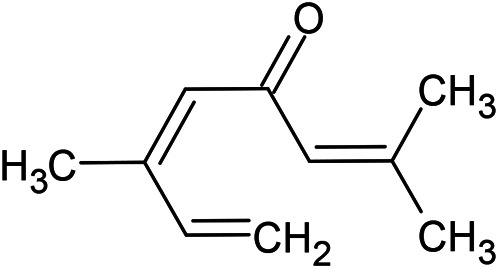						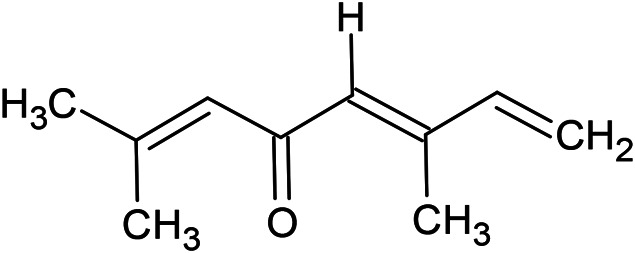				
α-Eudesmol			(Z)-Ocimenone						(E)-Ocimenone				
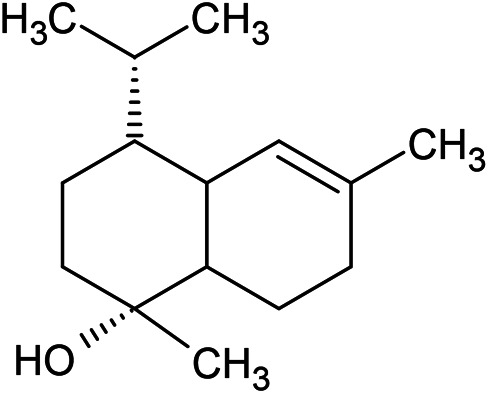			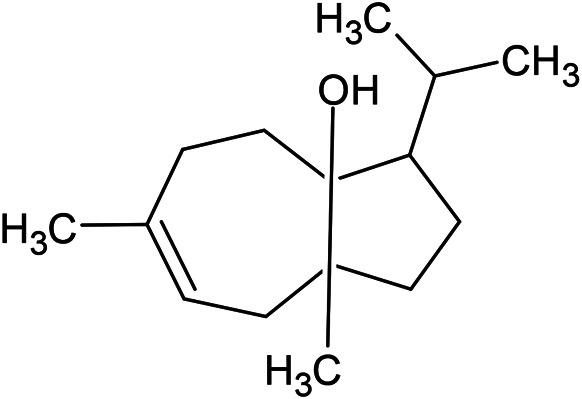						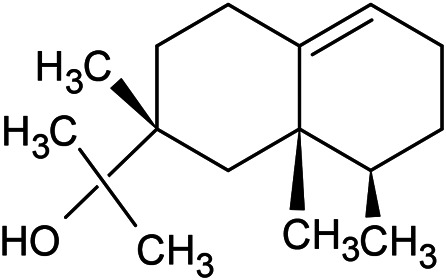				
Epi-α-muurolol			Carotol						Valerianol				
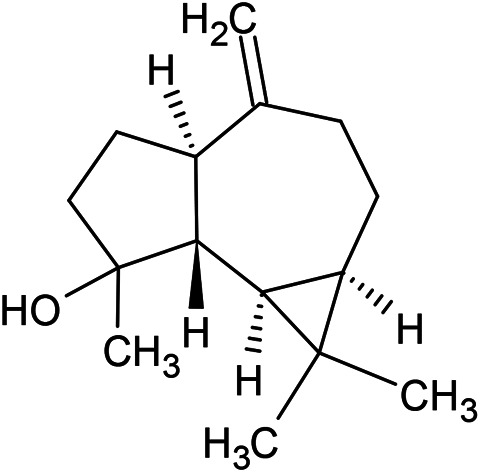			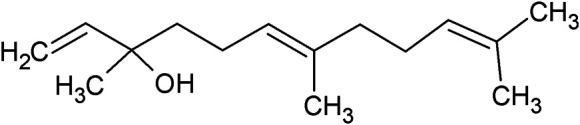						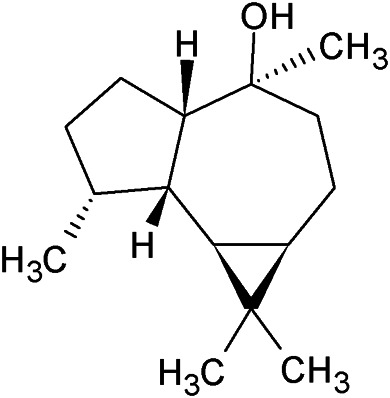				
Spathulenol			(E)-Nerolidol						Viridiflorol				
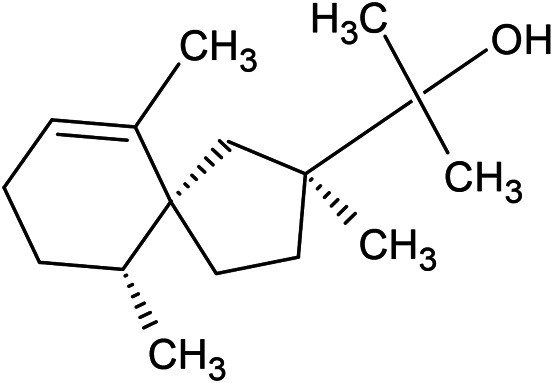													
Hinesol													
**Oxygenated sesquiterpenoids**													
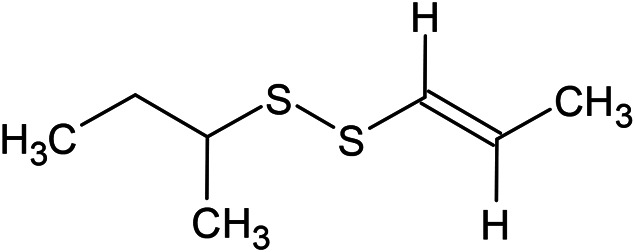				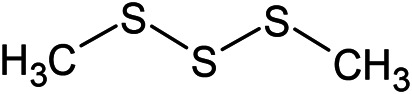				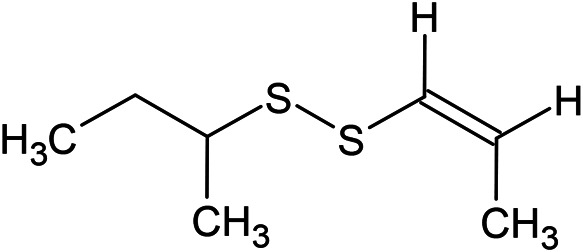					
(E)-1-Propenyl-sec-butyl disulfide				Dimethyl-trisulfide				(Z)-1-Propenyl sec-butyl disulfide					
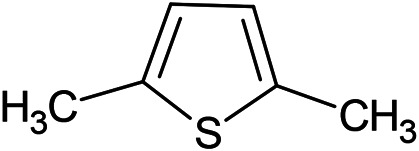				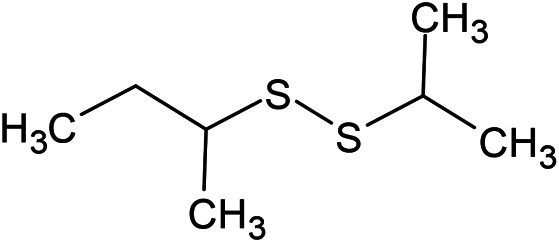				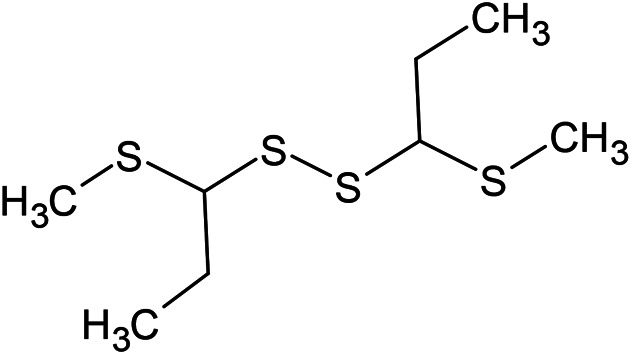					
2,5-Diethylthiophene				Di-sec-butyl-disulfide				Bis-[(1-methylthio) propyl]-disulfide					
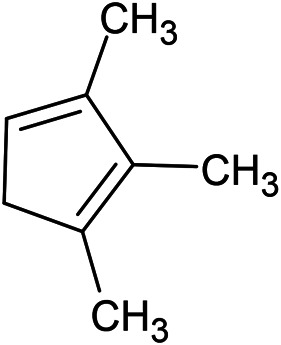													
Trimethylthiophene													
**Sulfur-containing compounds**													

The most commonly used essential oil extraction methods are steam distillation and hydro-distillation which are archaic methods that have been around for a long time (Mohammadhosseini, 2016). However, since the late twentieth century, microwave methods, such as solvent-free microwave extraction along with microwave-assisted hydrodistillation have been used for a faster and more effective extraction of plant essential oils (Mohammadhosseini and Nekoei, 2014; Meena et al., 2016; Meena et al., 2017c; Mohammadhosseini et al., 2017; Ragab et al., 2019). Akhzari and Saadatfar (2019) demonstrated the effect of stress conditions on the phytochemistry of essential oil of *Ferula haussknechtii* H. Wolff ex Rech. f*.* The result showed that the addition of lead nitrate (2 mM) decreased the myrcene, *α*-, and *β*-pinene concentration in the essential oil. The relation between the time of material collection (months) and the chemical composition of essential oil obtained from the oleo-gum-resin of *F. assa-foetida*, was evaluated by Kavoosi and Rowshan (2013). The oleo-gum-resins (OGRs) collected in three different collection times namely OGR1, OGR2, and OGR3 showed different essential oil chemical compositions (E)-1-propenyl sec-butyl disulfide (23.9%) and 10-epi-c-eudesmol (15.1%) were the major constituents in OGR1; whereas (Z)-1-propenyl sec-butyl disulfide and (E)-1-propenyl sec-butyl disulfide with a 27.7% and 20.3% quantity in OGR3; *β*-pinene (47.1%) and *α*-pinene (21.3%) were the major constituents in OGR2, respectively. Moreover, Karimian et al., (2020) observed a relationship between the collection time and the essential oil composition and quantity. The OGRs collected in July, August, September, and October showed 9.1%, 8.2%, 7.8%, and 7.4% oil content, respectively. The chemical compositions of the essential oil also varied in different age groups of plants. The essential oil of the young *Ferula assa-foetida* H. Karst. plant showed a higher amount of sulfur-containing and sesquiterpene compounds, whereas the essential oil of older plants displayed monoterpene (camphene) as the main chemical group of the essential oil profile (Mohammadi et al., 2019). Khalifaev et al., (2018) revealed the chemical profile of the *Ferula kuhistanica* Korovin essential oil using plants from two different locations of the central part of Tajikistan. The gas chromatography—flame ionization detector and gas chromatography-tandem mass spectrometry analysis of the hydrodistilled root oil, showed that monoterpene hydrocarbons (86.7%) had a greater proportion in oil; *a*-pinene (57.7–70.6%) was an abundant monoterpene hydrocarbon, and others include *ß*-pinene, *ß*-phellandrene and myrcene. Sagyndykova et al., (2019) identified 47 chemical components in the *Ferula foetida* (Bunge) Regel essential oil, using the GC-MS analysis. The samples were taken from two different populations (Tuyesu sands and Tynymbay Shoky hills) of the Mangyshlak peninsula which grew in two different types of soil (loamy and sandy). Both of the aforementioned populations were found to have respectable types of chemical components which were as follows; 2,5-dimethyl-2,5-dimethyl-thiophene, 3,4-dimethylthiophene, guaiol, myristicine, bulnesol, *α*-pinene, caryophyllene oxide, 2,5-dipropylthiophene, elemicine, 1-heptatriacontanol, *β*-*trans-*caryophyllene, *β*-*cis*-caryophyllene, *α*-caryophyllene, *β*-pinene, dimethyl trisulfide, *α*-eudesmol, *β*-eudesmol, *β*-eudesmene and 2-ethylthieno [3.2-b]thiophene, 5,5-dimethyl-4-[(1E)-3-methyl-1,3-butadienyl]-1-oxaspiro [2.5]octane, S-9-thiabicyclo [3.3.1]non-6-en-2-yl. Asilbekova et al., (2019) assessed the chemical constituents of *Ferula kuhistanica*'s essential oil with their enantiomeric excesses. The results of enantioselective and gas chromatography-tandem mass spectrometry analysis of the essential oil showed compounds with enantiomeric excesses as follows: (+/–)-*α*-pinene (61.9/38.1), (+/–)-*β*-pinene (28.6/71.4), (+/–)-sabinene (13.2/86.8), and (+/–)-limonene (82.7/17.3). The results of the study on the essential oils of *Ferula aucheri* Boiss. Piwczynski, Spalik, M. Panahi & Puchalka revealed the abundance of sesquiterpene hydrocarbons. The main components of flowering tops oil were germacrene B and *β*-caryophyllene (14.96 and 12.87%, respectively) and neophytadiene (diterpene compound, 0.18%), whereas *cis*-dihydroagarofuran and *δ*-cadinene (9.02 and 8.28%, respectively); *δ*-cadinene and gurjunene were found in fruit and root oils (18.25 and 12.62%, respectively) (Ahmadi et al., 2020). In a study, Kasaian et al., (2016) reported an abundance of oxygenated sesquiterpenes (74.7%) and S-containing hydrocarbons (16.6%) in the hydrodistilled essential oil of *Ferula alliacea* Boiss. The major components were 10-epi-*γ*-eudesmol, valerianol, hinesol, guaiol, and Z-propenyl-sec-butyl trisulphide with 22.3, 12.5, 8.3, 7.3, and 6.5% quantities, respectively. Malekzadeh et al., (2018) assessed the difference in yield and chemical components of the *Ferula gummosa* Boiss. Essential oils were growing in different types of bioclimates. The galbanum dry weight vs. essential oil amount was found to be the highest in the plants collected from Ebrahim Abad (16.9%) and the lowest in the plants collected from angouran (11%). *F. gummosa* oil contained a higher number of monoterpenes hydrocarbons such as 17–56.55% of *α*-pinene, 10.44–37.04% of *β*-pinene, 9.16–10.75% of *δ*-3-carene, and 0–13.23% of Limonene, moreover, a chemo-variation was observed in the oils. Kouyakhi et al., (2008) described that the plant samples (*Ferula gummosa*) collected from the tropical zone had a greater number and better type of flavoring combinations of essential oils as compared to the samples collected from the cold region. The samples collected from the same region, but at different altitudes could affect the chemical profile of a plant's essential oil and reflects the efficacy of environmental effects. Moreover, the samples collected from the different regions and at the same altitude also showed variation in phytochemicals. Using an environmental metabolomics approach, the effect of edaphic factors (pH, texture, and iron, aluminum, and potassium content) and environmental factors (temperature latitude, altitude and longitude) were determined using the essential oils from the roots of 10 Iranian *F. assa*-*foetida*. Three different types of plant chemotypes were characterized with different major chemical compounds in their essential oils. A chemotype (I) possessed Z-1-propenyl sec-butyl disulfide and monoterpenes; *α*-agarofuran and eudesmane sesquiterpenoids by chemotype (II); and Z-1-propenyl sec-butyl and E-1-propenyl sec-butyl by chemotype (III) Karimi et al., (2020). Fourteen Iranian and Afghan *F. assa-foetida* L. were investigated for their essential oil composition hydrodistilled from oleo gum-resin using a Clevenger type apparatus; a range of variations among 42 compounds was reported. The major constituent was (E)-1-propenyl sec-butyl disulfide (13.66–49.35%), *β*-pinene (Z)-1-propenyl sec-butyl disulfide, *α*-pinene, thiophene, and thiourea. The results of the analysis showed that the increase in altitude caused an increase in *β*-pinene and (Z)-1-propenyl sec-butyl disulfide content, but a reduction in the thiourea content in essential oils. This study showed that altitude was the environmental factor exerting the greatest effect and which caused a great number of variations in the essential oils' chemical components and yield (Hassanabadi et al., 2019). *Ferula* has many uses in ethnobotany due to its vast phytochemistry but the use of its essential oil has not been seen so much in folk and traditional medicine. Nevertheless, the volatile oils present in the gum of *Ferula assa-foetida* are released from the body through the lungs, thus, its essential oil is an excellent treatment for asthma. Apart from this, it is also very useful in discharge, breathing, flatus, and gastric erosions (Mahendra and Bisht, 2012).

## Bioactivities of the Essential Oils From the *Ferula* Species

The essential oil extracted from the *Ferula* species display different types of biological activities. These activities are related to the chemical group present in the essential oils. These bioactivities could be connected to a single compound or group of chemical compounds found in the essential oil. All-important biological activities and major chemical constituents of the essential oils from different species of the *Ferula* genus are shown in Table 2. These bioactivities of essential oils are discussed below.

**TABLE 2:** Tested living system, different bioactivities and major chemical components of the essential oil from different species of *Ferula* genus growing in different countries.

Plant name (province)	Used plant part (extraction method); essential oils major components	Activity	Tested living system	Result	References
*Ferula assa-foetida* L. (Iran)	Gum (HD)	Cytotoxic	Breast cancer 4T1 cells	Result showed that all constituents of essential oil could inhibit 4T1 cell proliferation in time and dose dependent manner	Bagheri et al., (2017a)
	Seeds (soxhlet)	Antinociceptive	Male albino mice	Exhibited valuable antinociceptive activity on acute and chronic pain in mice	Bagheri et al., (2017b)
		Relaxant	Male wistar rats ileum	Seed and assa-foetida essential oils (0.3% and 0.2%) could be significantly decrease ach (10^−4^ M) induced contractions by 35.6 (4.12) and 8 (2.4), *p* = 0.03; 43% and 12%, *p* = 0.02, respectively	Bagheri et al., (2014a)
	Gum (HD); 1-(2-methyl-1,3-oxathiolan-2-yl) ethanone, (thiophene, *trans*-dibenzylideneacetone and (Z)-propenyl sec butyl disulfide	Vasodilatory	Rats' thoracic aorta	AEO showed a significant vasodilatory effect which could be endothelium-independent or dependently	Esmaeili et al., (2017)
	Oleo-gum resin (HD)	Vasorelaxant	Rats' thoracic aorta	AEO displayed relaxant effect on the precontracted rings, dose-dependently at 23 μL/L IC_50_ value	Esmaeili et al., (2020)
		Antiprotozoan	*Blastocystis* sps	The lowest and highest percentage inhibition value at which *Blastocystis* showed no growth and multiplication were 16 and 40 mg/ml	El Deeb et al., (2012)
	Oleo-gum-resin (HD); OGR1: 10-Epi-c-eudesmol and (E)-1-propenyl sec-butyl disulfide; OGR2: (E)-1-propenyl sec-butyl disulfide (Z)-1-propenyl secbutyl disulfide; OGR3: *α*-pinene and.*β*-pinene	Antimicrobial	Gram-negative bacteria: *Escherichia coli* PTCC 1330 and *Salmonella typhi* PTCC 1609; foodborne gram-positive bacteria *Bacillus subtilis* PTCC 1023 and *Staphylococcus aureus* PTCC 1112; foodborne fungi: *Candida albicans* PTCC 5027 and *Aspergillus niger* PTCC 5010	MIC (minimal inhibitory concentration) for gram-negative and positive bacteria and fungi were 0.018–0.058, 0.027–0.107 and 0.028–0.111 mg/ml of essential oil, obtained from OGR3, OGR2 and OGR1, respectively	Kavoosi and Rowshan (2013), Kavoosi et al., (2015)
	Air-dried leaves latex (HD); E-1-propenyl-sec-butyl disulfide, *β*-pinene and *β*-ocimene	Scolicidal	*Echinococcus granulosus*	Results indicated that 10 min exposure of *F. assafoetida* essential oil with 60 μg/ml or more concentration were killed all protoscolices	Kavoosi et al., (2013)
	Herbaceous plant (HD)	Insecticidal	*Ectomyelois ceratoniae*	All concentration showed complete inhibition of pest	Goldansaz et al., (2012)
	Dried aerial parts (HD)	Insecticidal	*Callosobruchus maculatus*	*F. assafoetida* L (150 uL) showed 100% mortality against adult *C. maculatus*, respectively	Estekhdami et al., (2020)
	Gum (HD); (E)-1-propenyl sec-butyl disulfide, *β*-pinene, (E)-*β*-ocimene, (Z)-*β*-ocimene and *α*-pinene	Insecticidal	*Aphis gossypii*	Exhibited appreciable insecticidal activity at 0.04 μL/L LC_50_value and repellant activity at μL/mL	Koorki et al., (2018)
	Gum (HD)	Insecticidal	*Rhyzopertha dominica*	Essential oil showed toxicity against *R*. *dominica* with 65.71 h persistence	Bahrami et al., (2016)
	Latex (HD)	Insecticidal	*Agonoscena pistaciae*	LC_50_ values of essential oil were obtained 5.62 mg/L	Izadi et al., (2012)
	Foliar parts (HD)	Anti-quorum sensing	*Pseudomonas aeruginosa Chromobacterium violaceum*	Essential oil caused reduction in pyocyanin, pyoyerdine, elastase, biofilm and homoserin lactones (HSL) production in *p*.* aeruginosa* and also inhibited violacein production in *C*. *violaceum*	Sepahi et al., (2015)
	Gum (HD); (E)-1-propenyl sec-butyl disulfide, (Z)-1-propenyl sec-butyl disulfide and 10-epi-γ-eudesmol	Antibiofilm	*Candida albicans*, *C. dubliniensis*, *C. krusei*	4 μL/ml concentration of tested essential oil completely inhibited biofilm formation	Zomorodian et al., (2018)
	Seeds and oleo-gum-resin (HD); (E)-1-propenyl sec-butyl disulfide and (Z)-1-propenyl sec-butyl disulfide	Antimicrobial	*Lactobacillus rhamnosus*, *Streptococcus sobrinus*, *S. mutans*, *S. salivarius* and *S. sanguis*	The -resin oil had strongest antibacterial properties than the seed oil (*p* < 0.001)	Daneshkazemi et al., (2019)
	Resin (HD); guaiol (13.66%) and *β*-Pinene	Antibacterial	*β*-Lactamase producing *Acinetobacter baumannii*	Showed antibacterial effect with 18.75 mg/ml MIC value	Afshar et al., (2016)
	Dried plant materials (HD)	Antifungal	*Penicillium digitatum* and *P. italicum*	The essential oils showed growth inhibition along with conidia germination and germ tube elongation inhibition	Zahani and Khaledi (2018)
*Ferula assa-foetida* L. (India)	Stem and root (HD); (E) and (Z) sec-butyl propenyl disulfide and methyl 1-(methylthio) propyl	Ovicidal and larvicidal	Larvae of *Culex pipiens and Culex restuans*	Result showed *Culex restuans* (LC_50_: 10.1 mg/L) more sensitive to essential oil than *Cx. Pipiens.* The eggs exposed to essential oils were (55.8%) failed to hatch	Muturi et al., (2018)
	Latex (HD)	Anti-quorum sensing	*Chromobacterium violaceum*	Essential oil reduced the production of violacein and pyocyanin in *C. violaceum* and *P*. *aeruginosa*, respectively	Khambhala et al., (2016)
	Latex (HD); *β*-pinene, and 1,2-dithiolane and *α*-pinene	Antifungal and antibacterial	*Salmonella typhi*, ***E*** *. coli*, *S. aureus*, ***B*** *. subtilis*, ***A*** *. niger*, and ***C*** *. albicans*	MIC values 90 ± 11, 85 ± 5, 80 ± 12, 125 ± 17, *>*200, and *>*200, μg/mL were reported for *S. typhi*, ***E*** *. coli*, ***C*** *. albicans*, *S. aureus*, ***B*** *. subtilis*, ***A*** *. niger*, *S. aureus* and ***B*** *. subtilis*, respectively	Kavoosi et al., (2013)
	Gum (HD); pathani: 1-(1- propenylthio) propyl methyl disulfide and (E)-1- propenyl sec butyl disulphide; irani: (E)-1- propenyl sec-butyl disulfide and (Z)-1- propenyl sec-butyl disulfide	Antifungal and antibacterial	*Aspergillus niger*, ***A*** *. flavus*, ***A*** *. ochraceus*, *Fusarium oxysporum*, *P. chrysogenum*, *S. aureus*, *Yersinia enterocolitica*, *Salmonella paratyphi*, *S. typhi*, *Bacillus subtilis*, ***B*** *. cereus*, *Escherichia coli* and *Listeria monocytogenes*	Essential oil from pathani and irani *F. assa-foetida* exhibited a good antibacterial (*Bacillus subtilis* and *Escherichia coli*) and antifungal (*Aspergillus ochraceus* and *Penicillium chrysogenum*) activity, respectively	Divya et al., (2014)
	Resin (soxhlet)	Antifungal	*Alternaria alternata*, *A. solani*, *A. flavus*, *A. niger*, *A. wentii*, *Rhizoctonia* spp. *Drechslera tetramera*, *D. hawaiiensis*, *Fusarium semitectum*, *F. moniliforme* and *F. solani*: Isolated from okra seeds	Essential oil exhibited significant inhibiton at 0.25% of essential oil concentration	Sitara et al., (2018)
*Ferula aucheri* boiss. Piwczynski, spalik, M. Panahi and puchalka (Iran)	Dried flowering tops, fruits, and roots (HD); *β*-caryophyllene and germacrene B (roots); *δ*-cadinene (flowering tops); gurjunene and *δ*-cadinene (fruit)	Antimicrobial	*Pseudomonas aeruginosa*, *E. coli*, *Staphylococcus epidermidis*, *S. aureus*, *Bacillus subtilis*, *K. pneumonia*, *Shigella dysenteriae*, *Salmonella paratyphi*, *Proteus vulgaris*, *Candida albican, Aspergillus brasiliensis* and *A. niger*	Exhibited lower MICs value for *Klebsiella pneumonia*, *Bacillus subtilis*, *Salmonella paratyphi*-A serotype and *Shigella dysenteriae* compared to gentamicin, whereas fruit and root oils were most efficient against *E. coli* compared to gentamicin	Ahmadi et al., (2020)
*Ferula assafoetida* L. (kakan)	Leaves and seeds (HD)	Insecticidal	Black bean aphid	Highest death (13.5) rate in essential oil treated black bean aphid was reported than the control (1.5)	Bagheri and Rahimi (2014)
*Ferula communis* L. (Tunisia)	Aerial parts (HD); *β*-caryophyllene, *β*-myrcene, *α*-pinene, *α*-eudesmol, γ-curcumene, *p*-menth*α*-1,5-dien-8-ol and γ-eudesmol	Antileishmanial	*Leishmania infantum* and *Leishmania* (L.) *major* promastigotes	Caryophyllene had high leishmaniacidal activity against *L. major* (1.33 ± 0.52 μg/ml) and *L. infantum* (1.06 ± 0.37 μg/ml)	Essid et al., (2015)
	Flowers, leaves, stems, and roots (HD); camphor, *β*-eudesmol, and *α*-pinene (flower); *β*-eudesmol, aeudesmol and *δ*-eudesmol (Stems); dillapiole, guaiol and spathulenol (roots); *α*-eudesmol, deudesmol and *β*-eudesmol (leaf)	Antibacterial	*Pseudomonas aeruginosa*	The best results were showed by the essential oil of leaves against *P. aeruginosa* (MIC value 0.156 mg/ml)	Nguir et al., (2016)
*Ferula communis* L. (Turkey)	Aerial parts (HD)	Antibacterial	*Chryseobacterium indologenes*	Result showed the essential oil had a great potential to control *C. indologenes*	Dadaşoğlu et al.,(2018)
*Ferula cupularis* boiss. (Iran)	Flower leaves stem (HD); *δ*-2-carene (flower); *β*-ocimene and *β*-pinene (leaf); *δ*-3-carene and *α*-terpinyl isobutyrate (stem)	Antibacterial	*Staphylococcus epidermidis*, *S. aureus*, ***B*** *. subtilis*, ***E*** *. coli*, ***K*** *. pneumonia*	Showed 22.75, 5.69 and 2.85 mg/ml MIC value of flower, leaf and stem (except *P. aeruginosa*) essential oils against tested gram-positive bacteria	Alipour et al., (2015)
*Ferula elaeochytris* korovin (France)	Fruits (HD); *α*-pinene, *β*-phellandrene and sabinene	Antibacterial and antifungal	*S. aureus*, *E. coli*, yeast, *C. parapsilosis*, *C. albicans*, *Cryptococcus neoformans*, *T. violaceum*, *T. soudanense*, *T. rubrum*, *T. mentagrophytes*, *T. tonsurans*, *A. spergillus* and *A. fumigatus*	Presented the highest inhibition against *Staphylococcus aureus Trichophyton* species	Khoury et al., (2018)
*Ferula ferulaeoides* (steud.) korov. (China)	Root (HD)	Insecticidal	*Plutella xylostella*, *Mythimna separate* and *Musca domestica*	Guaiol showed great contact inhibition of *Plutella xylostella* and *Mythimna separate* at LD_50_ values: 8.9 and 0.07 mg/larva, respectively whereas, fumigation activity against the *M. domestica* and *M. separata*, were observed at LC_50_ values of 16.9 μL/L and 3.5 μL/L, respectively	Liu et al., (2013)
*Ferula galbaniflua* boiss. and buhse. (Brazil)	Leaves and stems (HD); methyl-8-pimaren-18-oate and ethyl phthalate	Antiprotozoan	*Leishmania amazonensis*	Essential oil had potent effect on against *Leishmania amazonensis* and its IC_50_/24 h value was 54.05–162.25 μg/ml	Andrade et al., (2016)
*Ferula gummosa* boiss. (Iran)	Gum (HD); *α*-pinene, carvacrol methyl ether, *δ*-3-carene and *β*-phellandrene	Cytotoxic	L929 mouse fibroblast cells	The average cell viability of L929 cells were observed about 88% at full concentration (50 μL/ml) of the essential oil	Abbaszadegan et al., (2015)
	Gum (market product)	Hypoglycemic and hypolipidemic	Male wistar rats	Glucose, triglyceride, cholesterol LDL-C and HDL-C IC_50_values for *F. gummosa* were recorded as 429.91 ± 46.14, 105.18 ± 12.13, 91.02 ± 11.95, 29.59 ± 3.76, 42.07 ± 9.68 (mg/dl), respectively	Karimlar et al., (2019)
	Market product	Insecticidal	*Bemisia tabaci* and *Orius albidipennis*	For *Bemisia tabaci*, 0.059 uL^−1^ LD_50_ value was observed along with significant effect against *O*. *albidipennis*	Zandi-Sohani et al., (2018)
	Gum (HD)	Insecticidal	*Callosobruchus maculatus*	The essential oil showed 100% death rate at 500 μL/L after 6 h exposure interval	Hosseinpour et al., (2011)
	Root (HD)	Insecticidal	*Ephestia kuehniella*	Topical application presented drastic reduction in total hemocyte count	Ghasemi et al., (2014)
	Aerial part (HD)	Antibacterial	*Enterococcus faecalis*, *Streptococcus sobrinus*, *S. salivarius* and *S. mutans*	The MIC value 1.0125 μg/ml was reported for *E. faecalis* and other strains	Nazemisalman et al., (2018)
	Resin (market product)	Antibacterial	*Staphylococcus aureus*	Showed the best antibacterial activity against methicillin resistant *S. aureus* (MRSA) and methicillin sensitive (MSSA) bacterial strains	Mahboubi et al., (2011)
	Resin (HD)	Antibacterial	*Pseudomonas aeruginosa*	Revealed the significant antibacterial activity of plant essential oils and extracts (*p* < 0.001)	Satarian et al., (2018)
	Resin (HD); *α*- pinene, *β*-pinene and *δ*-3-	Acaricidal	*Tetranychus urticae*	Essential oil displayed high toxicity on adults and eggs of *T. urticae* with LC_50_ value 6.52 and 6.98 μL/L, respectively	Fatemikia et al., (2017)
*Ferula haussknechtii* H. Wolff ex rech. f. (Iran)	Root and aerial parts (HD); camphene, isoverbanol and *α*-pinene	Antibacterial	*Bacillus cereus*, ***B*** *. subtilis*, ***B*** *. pumilus*, ***E*** *. coli*, *S. epidermidis*, ***K*** *. pneumonia*, *S. aureus*, ***E*** *. faecalis* and *p. aeruginosa*	Essential oil exhibited efficient activity against *Staphylococcus aureus*, *Staphylococcus epidermidis* and *Bacillus pumilus* at 7.5 mg/ml concentration; 17–18 mm growth inhibition	Arjmand and Dastan (2020)
*Ferula hermonis* boiss. (France)	Root and rhizome (HD); *α*-bisabolol, *δ*-cadinene and. *β*-farnesene	Antifungal	*Aspergillus fumigatus*, *A. niger*, *Candida albicans*, *Microsporum gypseum*, *Penicillium purpurogenum*, *Saccharomycescerevisiae* and*Trichophyton mentagrophytes*	Essential oil exhibited the strongest antifungal activity against *T. mentagrophytes*	Al-Ja'fari et al., (2011)
*Ferula heuffelii* griseb. Ex heuff. (Serbia)	Underground part (HD); elemicin, phenyl propanoids, myristicin and *α*-pinene	Antibacterial and anticandidal	Gram-positive bacteria: *Staphylococcus aureus*, *S. epidermidis*, *Micrococcus luteus*, *Bacillus subtilus* and ***B*** *. cereus*; gram*-*negative bacteria: *Salmonella typhimurium*, *Pseudomonas aeruginosa* and *Escherichia coli*; fungi: *Candida glabrata*, *C albicans*, ***C*** *. tropicalis* and ***C*** *. sake*	Displayed antimicrobial potential against two strains of *Micrococcus luteus*, *Staphylococcus epidermidis*, *Micrococcus flavus* and *Candida albicans*; MIC value 13.7, 13.7, 17.6, 21.1, 28.2 and 7.0 μg/ml, respectively	Juglal et al., (2002), Pavlović et al., (2012)
*Ferula lycia* boiss. (Turkey)	*α*-pinene, bornyl acetate, limonene and *β*-Pinene	Antibacterial	*Staphylococcus epidermidis*, *S. aureus*, *Enterococcus faecalis*, *E. cloacae*, *Klebsiella pneumonia*, *Escherichia coli*, *Serratia marcescens*, *Salmonella typhimurium*, *Proteus vulgaris*, *Haemophilus influenza* and *Pseudomonas aeruginosa*	*Haemophilus influenzae* ATCC 49247 was reported as the most sensitive bacteria among all tested one with 14 mm inhibition zones	Kose et al., (2010)
*Ferula lutea* (poir.) maire. (Tunisia)	Roots (HD); delta-3-carene, *α*-pinene, *β*-myrcene and *α*-phellandrene	Cytotoxic	Human colon cells (HT-29 and HCT-116 cells)	The IC_50_ values were 26.39 ± 3.98 μg/ml and 81.00 ± 12.81 μg/ml for HT-29 and HCT-116, respectively	Ben et al., (2016)
*Ferula lutea* (poir.) maire. (France)	Roots (HD); delta-3-carene, alpha-phellandrene, myrcene and *α*-pinene -	Antibacterial	*Staphylococcus epidermidis, S. aureus*, *Micrococcus luteus*, *Bacillus cereus*, ***B*** *. subtilus*, ***E*** *. coli*, *P. aeruginosa*, *Salmonella typhimurium*, *Candida albicans*, ***C*** *. glabrata* ***C*** *. tropicalis*	The essential oil exhibited antibacterial and anticandidal activity against *E. coli*, *Salmonella typhimurium*, *Candida albicans* with MIC value; 39, 78 and 156 mg/ml, respectively	Ben et al., (2016)
	Flower (HD); delta-3-carene, 2, 3, 6-trimethylbenzaldehyde and *α*-pinene	Antibacterial and anticandidal	Gram-negative bacteria: *Salmonella typhimurium Pseudomonas aeruginosa* and *Escherichia coli*; gram-positive bacteria: *Staphylococcus aureus*, *S. epidermidis Micrococcus luteus*, *Bacillus subtilis* and ***B*** *. cereus*;*Candida glabrata*, ***C*** *. albicans*, ***C*** *. tropicalis* and ***C*** *. sake*	Exhibited antibacterial and anticandidal activity against *S. epidermidis*, *S. aureus* and *E. coli* (MIC = 39 μg/ml); and *C. albicans* (MIC = 156 μg/ml)	Znati et al., (2012)
*Ferula orientalis* L. (Turkey)	Stem (soxhlet); *α*-pinene, terpinolene, *o*-cymene, *β*-caryophyllene, limonene and bornyl acetate	Neuroprotective	Cortex neuron cells	Essential oil at 10^−2^ concentration showed significant neuroprotective activity	Topdas et al., (2020)
	Aerial parts (HD); *α* and *β*-Pinene	Antimicrobial	*Staphylococcus aureus*, *Escherichia coli*, *Pseudomonas aeruginosa* and *Candida albicans*	The oil of *F. orientalis* aerial parts was active against *Candida albicans* and *Staphylococcus aureus* strains	Karakaya et al., (2019a)
*Ferula ovina* (boiss.) boiss. (Iran)	Aerial parts (HD); *α*-pinene, camphene, sabinene, *β*-pinene, sabinene, myrcene and dehydro-1, 8-cineole	Antinociceptive and hyperalgesia	Mice and other animals	Essential oil constituents impart both antinociceptive and hyperalgesic potentially	Radulović et al., (2013)
	Dried root (HD)	Insecticidal	*Sesamia cretica*	Each concentration found to have significant effect on the immune ability of *Sesamia cretica*	Sadeghi et al., (2017)
*Ferula persica* willd. (Iran)	Gum (HD); alpha-pinene, (Z)-1-propenyl sec-butyl disulfide *β*-pinene, *β*-dihydroagarofuran, allo-aromadendrene, (Z)-*β*-ocimene, *β*-dihydrobenzofuran, *α*-caryophyllene and (E)-1-propenyl sec-butyl disulfide	Cytotoxic	Vero cell, murine colon carcinoma (CT26) cell lines	The viability was decreased in essential oil treatedVero cell (0.125 × 10^−9^ to 80 μL/ml) and CT26 cell line (0.125 × 10^−9^ to 20 μL/ml), dose dependently	Hosseinzadeh et al., (2020a), Hosseinzadeh et al., (2020b)
*Ferula tadshikorum* pimenov. (Tajikistan)	Underground parts (HD); (Z)-sec-butyl propenyl disulfide, (E)- sec-butyl propenyl disulfide and (E)-1-propenyl 1-(methylthio)propyl disulfide	Cytotoxic	CEM/ADR5000 and CCRF-CEM cell lines	Displayed 142.5 and 21.6 μg/ml, IC_50_ values for CEM/ADR5000 and CCRF-CEM cell lines, respectively	Sharopov et al., (2019)
	Underground (HD); (E)-1-propenyl sec-butyl disulfide, (Z)-1-propenyl sec-butyl disulfide, and (E)-1-propenyl 1-(methylthio)propyl disulfide	Antibacterial	Methicillin-resistant *Staphylococcus aureus*, *Escherichia coli*	Essential oil was not displayed any effective antibacterial activity up to 20 mg/ml concentration	Sharopov et al., (2019)
*Ferula tingitana* L. (Egypt)	Flowers and leaves (HD); *α*-thujene, eudesmol, elemol and cadinol	Antitumor	Hormone-responsive MCF7 breast cells, liver carcinoma (HePG2) cells, and cervical (HeLa) cells	IC_50_% value for *in vitro* cytotoxicity of flower and leaves essential oil against liver (HEPG2) carcinoma cell, cervical (HELA) and breast (MCF7), lines were as follows; 4.4, 4.2; 8.6, 10.9 and 6.9, 4.8, μg/mL, respectively	Elghwaji et al., (2017)
	Flower and leaf (HD); terpinolene and *α*-thujene in leaf and flower derived oil, respectively	Antimicrobial	Bacteria: *Staphylococcus aureus*, *Bacillus subtilis*, *Streptococcus faecalis*, *Escherichia coli*, *Pseudomonas aeruginosa* and *Neisseria gonorrhea*; fungi: *Candida albicans*, *Aspergillus flavus*	Showed most efficient antibacterial activity against *Neisseria gonorrhoeae* and *Bacillus subtilis* with 41.9 and 48.3% potency compared to tetracycline	Elghwaji et al., (2017)
*Ferula tunetana* pomel ex batt. (Tunisia)	Seed oil (HD); *α*-pinene, myrcene, (Z)-*β*-ocimene, *β*-pinene, *β*-phellandrene and limonene	Antigerminative	*Medicago sativa*, *Triticum aestivum* and *Lactuca sativa*	Germination of seedlings were completely inhibited at 10 mg/5 ml and 20 mg/5 ml concentration	Znati et al., (2017)
	Seed (HD); *α*-pinene, (Z)-*β*-ocimene and *β*-pinene	Antimicrobial	Gram (+) bacteria: *Staphyllococcus aureus* ATCC 25923 and CIP106510, *Bacillus subtilus* ATCC 6633, ***B*** *. cereus* ATCC 14579, ATCC 11778 and *M. luteus* NCIMB 8166; gram (-) bacteria: *Escherichia coli* ATCC 25922, ATCC 35218, *Salmonella typhimurium* ATCC 13311, LT2DT104; *Pseudomonas aeruginosa* ATCC 27853, *Candida albicans* ATCC 90028 and ***C*** *. glabrata* ATCC 90030	Displayed valuable antimicrobial activity against *S. typhimurium* LT2 DT104 and ***B*** *. cereus* ATCC 14579 (inhibition zone 16.2–1.0 and 15.8–1.0 mm, respectively)	Znati et al., (2017)
	Flower (HD); *α*-Pinene, epi-*α*-muurolol, *β*-chenopodiol and himachalol	Insecticidal	*Tribolium castaneum*	Essential oils fractions displayed 93% repellent activity	Baccari et al., (2020)
*Ferula vesceritensis* coss. and durieu ex trab. (Algeria)	Flowers and stems (HD); *α*-pinene, *β*-pinene, elixene *α*-phellandrene, aristolene and fenchylacetate	Antibacterial	*Pseudomonas aeruginosa*, *Enterobacter aerogenes*, *E. coli*, *S. aureus*, *Klebsiella pneumonia*, *Morganella morganii*	Showed potent inhibition of tested bacterial strains	Labed-Zouad et al., (2015)
*Ferula vesceritensis* coss. and durieu ex trab. (East of algiers)	Dried leaves (HD); shyobunol, (t-cadinol, *δ*-3-cadinene, *α*-cadinol and aristolene	Insecticidal	*Sitophilus oryzae*	Contact and inhalation methods showed potent insecticidal activity with (LD_50_ = 16.4 μL/ml, LD_90_ = 53.45 μL/ml values	Benchabane (2014)
*Ferula vesceritensis* coss. and durieu ex trab. (Algeria)	Dried aerial parts (HD); germacrene D, 5, 9-tetradecadiyne, *α*-bisabolene and farnesene	Antibacterial	*Escherichia coli*, *Staphylococcus aureus* and *Klebsiella pneumonia*	The essential oil presented appreciable growth inhibition of listed bacteria	Zellagui et al., (2012)

## Insecticidal Activity

According to the obtained results, it was concluded that terpenes had active insecticidal properties, particularly as fumigants. Baccari et al., (2020) reported that *Ferula tunetana* Pomel ex Batt. Essential oils had insecticidal activity against *Tribolium castaneum* Herbst. The essential oils used as fumigants showed 93% repellency and toxicity against *T. castaneum* at 161.89 μL L/L LD_50_ value. Bagheri and Rahimi (2014) observed that the *Ferula assa-foetida* H.Karst*.* essential oil had a valuable effect on the mortality rate of the black bean aphid at 1% probability level. The highest mortality rate (13.5) in aphids was reported at 300 μL/ml (leaf essential oil) and 500 μL/ml (seed essential oil) oil concentration, compared to the control (1.5). Furthermore, Goldansaz et al., (2012) evaluated the insecticidal activity of *F. assa-foetida* essential oil against carob moth (*Ectomyelois ceratoniae*) a polyphagous pest of pomegranate. All concentrations used in the experiment (oil: solvent, 1:1, 1:3, and 1:5), showed significant inhibition of insect with *p* < 0.001. In another study, Estekhdami et al., (2020) also evaluated the insecticidal activity of *F. assa-foetida* essential oil against *Callosobruchus maculatus* in an interval of 8, 24, and 48 h, respectively. *F*. *assa-foetida* essential oil was used with 0, 30, 60, 90, 120, and 150 μL concentration and a death rate reported at 8, 24, 48 h intervals. The mortality rate was observed to be 100% at a 30 µL concentration. Fumigant toxicity and persistence of the essential oil of *F*. *assa-foetida* with two others were investigated against adult insects (*Rhyzopertha dominica* F.). The result suggests that *F*. *assa-foetida* essential oil has more toxicity against *R*. *dominica* compared to other tested plants. The half-life of the essential oil of asafoetida was reported to be 65.71 h which is also greater than the others (Bahrami et al., 2016). Hosseinpour et al., (2011) investigated the fumigant insecticidal activity of two plants including galbanum (*F*. *gummosa*) essential oils extracted from the gum with a Clevenger apparatus. The fumigant insecticidal activity of essential oils was examined alongside adult *Callosobruchus maculatus* (1–7 days old) with different concentrations ranging from 7.1 to 57.1 μL/L air, at 27–28°C room temperature, 65–70% relative humidity and in dark conditions. Galbanum oil presented a 47.5%, 80%, and 100% mortality rate at 500 μL/L (air) after 2, 4, and 6 h exposure intervals, respectively.

Liu et al., (2013) evaluated an insecticidal sesquiterpene guaoil, found in the essential oils of many plants. To examine the insecticidal property of the compound, three insect larvae were used, namely *Mythimna separata* Walker, *Plutella xylostella* L., and *Musca domestica* L. An effective contact inhibition was observed in the 4th instar larvae of *Mythimna separata* and third instar larvae of *P. xylostella* at 0.07 and 8.9 mg/larva concentrations of guaoil while using the fumigation method, the growth inhibition was observed at 3.5 and 16.9 μL/L guaoil concentration, respectively. Koorki et al., (2018) tested *F. assa-foetida* essential oil along with two other medicinally important plants to find their toxicity against *Aphis gossypii* Glover which has caused economic losses in tested medicinal plants. Lethal concentrations (LC_50_) of the essential oil of *F. assa-foetida* were examined after 12 (9.04 μL/L air) and 24 (4.64 μL/L air) hours whereas at a 10 μL/ml concentration the essential oil showed repellent activity. The chemical composition of the essential oil was also evaluated by GC-MS and the major chemical components were (E)-sec-butyl propenyl disulfide (Z) and (E)-*β*-ocimene, *β* and *α*-pinene. The effect of essential oils from *F. assa-foetida* L. with some other plant species was studied on the growth and physical fitness of *Trichogramma embryophagum* (Hartig) and *Trichogramma evanescens* (West.)—parasitoids on the eggs of carob moths. Essential oils at 877 ppm (LC01) exhibited a significant reduction in longevity, wing normality, development, female's fecundity, the sex ratio of their progeny, and survivorship (Poorjavad et al., 2014).

## Antiquorum Sensing and Antibiofilm Activity

Sepahi et al., (2015) evaluated the potential of *F*. *assa-foetida* essential oil to inhibit the quorum sensing in *Pseudomonas aeruginosa*. The essential oil (25 mg/ml) presented anti-quorum activity and fully abolished pyocyanin, pyoyerdine, elastase, biofilm, and homoserine lactone (HSL) production. *F. assa-foetida* essential oil mediated *las* system inhibition was observed in *Chromobacterium violaceum.* Since the violacein production is related to the *las* system, violacein production inhibition was also observed in *F. assa-foetida* essential oil treated *C. violaceum*. The chemical profile of the essential oil extracted from the resin of *F. assa-foetida*, antibiofilm and antimicrobial activity were also investigated by Zomorodian et al., (2018). The main components of essential oil analyzed by the GC/MS method, contained 21.65% of (E)-sec-butyl propenyl disulfide and 19.5% of (10-epi-γ-eudesmol and 10.20% of 2-[(Z)-prop-1-enyl] disulfanyl] butane. The essential oil exhibited partial inhibition (50%) of the biofilm formation in the standard strains of *Candida krusei*, *C. tropicalis* and *C. albicans* at concentrations of 0.06, 0.125, and 0.25 μL/ml, respectively. However, the essential oil showed 100% inhibition of biofilm formation at 4 μL/ml of concentration.

## Antimicrobial Activity

It has been reported that the essential oils obtained from the root and rhizome of the *Ferula hermonis* Boiss. had a significant effect on many fungal strains such as dermatophytes namely *Trichophyton mentagrophytes* (Al-Ja’fari et al., 2011). The main chemical components of the essential oil analyzed by GC–MS and FID, and 13C NMR were *α*-bisabolol, *β*-farnesene, and *δ*-cadinene. The MIC and MFC values of essential oils for *T. mentagrophytes* were reported to be 8 μg/ml and 10.25 μg/ml for 3, 5-nonadiyne and JB73, respectively. In a study, the antibacterial activity of hydrodistilled essential oil from fresh stems (FS), fresh flowers (FF), and dry stems (DS), dry flowers (DF) of *Ferula vesceritensis* Coss. & Durieu ex Trab. were demonstrated. The essential oils possessed *α* and *β*-pinene, *α*-phellandrene as abundant chemical constituents along with a minute amount of caryophyllene oxide, aristolene, carotol, and elixene (Kim et al., 2004). Nine strains of foodborne and clinically isolated bacteria including *Escherichia coli*, *Morganella morganii*, *Klebsiella pneumonia*, *Pseudomonas aeruginosa*, *Staphylococcus aureus*, and *Enterobacter aerogenes* were used for the antibacterial activity analysis. The minimum inhibitory concentration (MIC) was reported at a concentration of 128 μg/ml against almost all foodborne pathogens and clinical isolates, whereas for the others the concentration was 16–80 μg/ml (Labed-Zouad et al., 2015). Satarian et al., (2018) also tested the extract and essential oils of *Ferula gummosa* for their antibacterial activity against clinically isolated from *P. aeruginosa*. In another study, bactericidal activity of essential oil from *F. assa-foetida* was reported by Kavoosi et al., (2013) together with some other activity including antioxidant, antiseptic, sedative, antispasmodic, analgesic, and carminative activity. The essential oils comprised pinene (β: 47.1%; α: 21.36%) and 1,2-dithiolane (18.6%) as main compounds which showed a minimum inhibitory value at the concentration of 90 ± 11, 85 ± 5, 80 ± 12, 125 ± 17, *>*200 and *>*200 μg/ml against the tested strains namely *Candida albicans*, *Aspergillus niger*, *Bacillus subtilis*, *Staphylococcus aureus*, *Escherichia coli*, and *Salmonella typhi*, respectively. The antifungal and antibacterial activity of the flower oil of *Ferula elaeochytris* Korovin along with some other plant species was demonstrated against some selected Gram-positive, Gram-negative bacteria and fungal strains (Khoury et al., 2018). The essential oil was found to have the most antibacterial activity against the *Staphylococcus aureus* and dermatophytes (*Trichophyton* species) with a 8–64 μg/ml MIC value. The *F. haussknechtii* essential oil, which was extracted by the Clevenger apparatus contained camphene, *α*-pinene, and isoverbanol as abundant chemical constituents; these compounds exhibited antibacterial activity against nine bacterial strains. The result showed that *Bacillus pumilus*, *Staphylococcus epidermidis* and *S. aureus* were more sensitive to the essential oil among other tested bacterial strains (Arjmand and Dastan, 2020). Alipour et al., (2015) enumerated the bactericidal activity of the essential oil acquired from stem, leaf, and flower parts of the *Ferula cupularis* Boiss. against some Gram-positive bacteria. The main components that were analyzed by the GC-MS analysis displayed a great variation. The MIC value of flower, leaf, and stem (except *P. aeruginosa*) oils were 22.75 mg/ml, 5.69 mg/ml, and 2.85 mg/ml against tested Gram-positive bacteria. *Ferula gummosa* oil showed antibacterial potential against *Enterococcus faecalis*, *Streptococcus sobrinus*, *S. salivarius*, *S. mutans* with a 1.0125 mg/ml MIC value (Nazemisalman et al., 2018). Divya et al., (2014) investigated the antimicrobial activity of essential oils from two plant varieties (Pathani and Irani) of *F. assa-foetida*, against various food-borne bacterial and fungal species. Different types of plant varieties also possessed variations in their oil chemical profiles. In accordance with the result, a conclusion was made that the essential oil of the Pathani *F. assa-foetida* variety exhibited a good antibacterial activity (*Escherichia coli* and *Bacillus subtilis*); whereas, the essential oil of Irani the *F. assa-foetida* variety exhibited good fungicidal activity (*Penicillium chrysogenum* and *Aspergillus ochraceus*). Furthermore, a study revealed the antimicrobial properties of the essential oil of the resin and seed of *F. assa-foetida* against various oral bacteria such as *Lactobacillus rhamnosus*, *Streptococcus salivarius*, *Streptococcus sanguis*, *Streptococcus sobrinus,* and *Streptococcus mutans* (Daneshkazemi et al., 2019). The resulting analysis displayed that the oleogum-resin essential oil had significant and effective antibacterial activities as compared to the seed essential oil (*p* < 0.001). Jahani et al., (2015) reported the antibacterial activity of gelatin nano-capsules formulated from the essential oil of *F. assa-foetida*. Mallahi et al., (2018) studied the potential of *Ferula* essential oil to increase the shelf-life of *Gerbera jamesonii* (Gerbera Daisy) flower by inhibiting the pathogenic bacteria. Ahmadi et al., (2020) tested *F. aucheri* essential oil for its antimicrobial potential. All aerial parts of the plant used to extract oil had different major chemical components. All essential oils exhibited lower MIC for *Salmonella paratyphi*-A serotype, *Shigella dysenteriae*, *Klebsiella pneumonia*, and *Bacillus subtilis* but gentamicin exhibit greater MIC than essential oil which was used as a positive control. Additionally, fruit and root oils showed more efficacy against *E. coli* compared to gentamicin. Zomorodian et al., (2018) uncovered the relationship between the antimicrobial activity and the presence of (E)-1-propenyl sec-butyl disulfide and (Z)-1-propenyl sec-butyl disulfide as the major chemical compounds in the oil. In the same way, many studies have reported the link between antimicrobial activity of the essential oils with a high content of sulfur compounds (Iranshahi et al., 2008; Kavoosi and Rowshan, 2013; Divya et al., 2014). In this regard, Divya et al., (2014) demonstrated the morphological changes in fungal and bacterial cells tested with the *F. assa-foetida* essential oil using scanning electron microscopy. The morphological changes were like damaged bacterial membranes, irregular branching in fungal hyphae and sporulation inhibition and disruption of the cytoplasmic membrane, which result in leakage of ions and electrolytes. Additionally, *a*-pinene and 10-epi-γ-eudesmol were also reported as active terpenoids against the broad range of microorganisms (Rivas da Silva et al., 2012). Terpenoids are highly lipophilic in nature, so they target cell membranes and cause toxicity through cell membrane integrity disruption (Kovač et al., 2015).

## Immunomodulatory Effect

Oüzek et al., (2017) elucidated the chemistry and immunomodulatory activity of the essential oil procured from the dried plant material of the *Ferula iliensis* Krasn. ex Korovin material by the hydro-distillation method with Clevenger apparatus (Z) and (E)-propenyl sec-butyl disulfide 23.4–45.0% and 15.7–39.4%, respectively were the major chemical constituents of the extracted plant essential oil. On the basis of the results of the experiments, it was concluded that the essential oil had the potential to stimulate [Ca^2+^] ion mobilization and production of reactive oxygen species in the murine bone marrow phagocytes and human neutrophils. Furthermore, the effect of essential oil could be reversed by using capsazepine a TrpV1 channel antagonist, in a dose-dependent manner. This result indicated that TrpV1 channels were the target site for the essential oil component, which was likely to be (Z)-sec-butyl propenyl disulfide, aver by the molecular modeling method using a known TrpV1 agonist. Sadeghi et al., (2017) studies the effect of *F. ovina* essential oil on the immune system of *Sesamia cretica* Ledereer. The results proved that the essential oil had a visual effect on the *S. cretica’s* immune system. Four main circulating hemocytes were identified in the fourth instar larvae which included oenocytoides, granulocytes (GRs), prohemocytes, and plasmatocytes. The 4th instar larvae were injected with 1 uL of each concentration of *F. ovina* oils (1,000, 2,500 and 7,000 ppm). The total number of hemocyte and GR count enhanced with 1,000 ppm concentration while decreased at 2,500 and 7,000 ppm concentration, dose-dependently. However, plasmatocyte numbers declined for all the treatment concentrations but more significantly with increased doses. However, the number of nodules and the phenol-oxidase activity was not affected by any tested essential oil concentration. Schepetkin et al., (2016) substantiated the effect of the *Ferula akitschkensis* B.Fedtsch. ex Koso-Pol. Essential oils on human neutrophils cells. Seed and stem oil possessed 4(10)-thujene, *α*- and *β*-pinene; 4-terpineol, eremophilene, 2-himalachene-7*β*-ol myristicin and (E)-6,10-dimethylundeca-5,9-dien-2-one as their primary components. The report analysis showed that the major component of umbels seeds namely *β*-pinene, 4(10)-thujene, γ-terpinene, (E)-6,10-dimethylundeca-5,9-dien-2-one, pichtosin, and (E)-non-2-enal stimulated calcium mobilization in neutrophils cells. Especially, geranylacetone and isobornyl acetate showed great potential with 7.6 ± 1.9 and 6.4 ± 1.7 μM EC_50_ value, respectively. Additionally, the treatment of neutrophil cells with the aforementioned components except (E)-2-nonenal, resulted in desensitization of the neutrophils due to fMLF (N-formyl-Met-Leu-Phe) and IL-8 (interleukin-8) induced calcium ions flux and inhibition of N-formyl-Met-Leu-Phe -induced chemotaxis in cells. The effect of these components on calcium ions flux in neutrophils could be inhibited by TRP channel blockers (transient receptor potential). A further study averred that geranylacetone was a TrpV1 agonist, and cause Ca^2+^ ions influx in TrpV1-transfected human embryonic kidney 293 cells (HEK293 cells); whereas myristicin was a TrpV1 antagonist, and inhibited fMLF and IL-8 mediated neutrophil [Ca^+2^]_i_ flux stimulation and abolished capsaicin (zostrix)-induced Ca^2+^ions influx in TrpV1-transfected human embryonic kidney 293 cells.

## Anti-Acetylcholinesterase, Anxiolytic and Antispasmodic Activity

Ahmadi et al., (2020) unearthed the anti-acetylcholinesterase activity of the essential oils of flowering tops, fruits, and roots of the *Ferula aucheri* Boiss. Piwczynski, Spalik, M. Panahi & Puchalka with antimicrobial activity. The biochemistry of the oils was analyzed by GC-MS method; AChE inhibitory potential was assessed by Ellman's method with slight modification. The major portion of the chemical constituents of the essential oils belonged to the sesquiterpene hydrocarbon group (61.9%). The essential oil of roots and fruits showed weak acetylcholinesterase (AChE) inhibitory potential with 239.69 ± 3.5 and 554.05 ± 4.65 μg/ml half-maximal inhibitory concentration (IC_50_) values, respectively whereas flowering tops essential oil presented moderate AChE inhibitory activity with 179.06 ± 4.3 μg/ml IC_50_ values. Znati et al., (2012) also inquired about the anti-acetylcholinesterase and antimicrobial properties of flower oil from *Ferula lutea* (Poir.) Maire. The chemical compounds of the essential oils were analyzed by GC-MS. The major portion of essential oil chemical constituents was covered with monoterpene hydrocarbon and sulfur-containing compounds. The result showed that the flower oil exhibits significant AChE inhibitory activity with a IC_50_ value of 70.25 ± 5.41 μg/ml. Deveci et al., (2018) examined the anticholinesterase activity along with antioxidant and anti-tyrosinase activity of *F. elaeochytris* Korovin essential oils. The result of GC-MS and GC-FID showed the presence of 21.3% of *β*-cubebene, 17.5% of caryophyllene oxide, and 14.9% of *β*-caryophyllene. The anti-cholinesterase activity was measured by Ellman's spectrophotometric method. The essential oil of *F. elaeochytris* exhibited the highest anticholinesterase and anti-tyrosinase activities. The anticholinesterase activity of the root's essential oil of *Ferula lutea* (Poir.) Maire. was also demonstrated by Ben et al., (2016). The constituents of hydrodistilled essential oils were analyzed by 13C-NMR, GC (FID) and GC-MS spectroscopy had a higher portion of monoterpene hydrocarbons viz. delta-car-3-ene (72.6%), alpha-pinene, myrcene, and alpha-phellandrene. The results indicated that this essential oil exhibited efficient antiacetylcholinesterase bioactivity with a IC_50_ value of 28.56 ± 1.87 μg/ml. The effects of *Ferula heuffelii* Griseb. ex Heuff. essential oil on contractions (KCl and ACh induced or spontaneous) were examined by Pavlović et al., (2012). The essential oil has the potential to inhibit spontaneous contraction in rat ileum, dose-dependently. The essential oil exerted half of the atropine (positive control) effect at a ED_50_ value (median effective dose) of 86.64 μg/ml. The acetylcholine mediated induction of contractions in ileum was inhibited at 75.00 μg/ml of the essential oil concentration, whereas at 250.00 μg/ml of essential oil concentration, the spasmodic effect of KCl (80 mM) was almost abolished. Sadraei et al., (2001) examined the effect of *F. gummosa* essential oil with other extracts (methanolic extracts, petrolic hydro-alcoholic, and etheric). *F. gummosa* essential oil (FGEO) inhibited the response to KCl (80 mM) at 10–360 μg/ml concentration whereas at 180 μg/ml, concentration almost terminated the response to KCl. Two components of essential oil namely *α* and *β*-pinene (10 ng/mL–1.3 μg/ml and 2–138 ng/ml, serially) inhibited the tonic contraction induced by KCl in a dose-dependent manner. These two components also inhibited the ACh (80 mM) induced contraction. *α*-Pinene (180 and 90 ng/ml), *β*-pinene (160, 80, 40, and 20 ng/ml) inhibited the acetylecholine induced contraction up to 45 ± 9.7%, and 95 ± 1.7% to 79 ± 7.7%; 0.8 ± 0.8%,11 ± 7.3, 33 ± 7.3 and 95 ± 2.3% to 84 ± 78.9%, sequentially (*p* < 0.05). The relaxant effect of oleo-gum-resin and seed oils of the *F. assa-foetida* on isolated rat's ileum was investigated by Bagheri et al., (2014a), Bagheri et al., (2014b), and Bagheri et al., (2014c). To reveal the relaxant effect of asafoetida resin and seed essential oil, the isolated ileum of rats treated with three doses, and isotonic contractions of the essential oil. The contractions of the specimen were induced by different doses (0.3, 0.2, and 0.1%) of asafoetida and essential oil. The amplitude of contraction was recorded before and after exposure to acetylcholine (ACh) cumulative concentration. Results showed that asafoetida (0.2% and 0.3% concentration) had an antispasmodic effect on acetylcholine-induced contraction. The essential oil also had effective antispasmodic activity against acetylcholine-induced contraction at concentrations 10^−12^–10^−2^ M of seed and asafoetida essential oils (0.2% and 0.3%). These oils could cause a significant reduction in acetylcholine (10^−4^ M) induced contractions at (4.12) and 8 (2.4) concentration, *p* = 0.03; up to 43% and 12%, *p* = 0.02, respectively. Batra et al., (2020) revealed the presence of triterpenoids with 32 other chemical components in the essential oil of *Ferula sumbul* Hook. roots. The essential oil (50 μL/kg) showed considerable anxiolytic activity in various tested models (light/dark, mirror chamber, elevated plus maze, *m*-CPP-induced and open-field anxiety). The results showed that the anxiolytic effect of the essential oil was mediated primarily through the benzodiazepines site on GABA receptors and through 5-HT receptors.

## Genotoxic and Antigenotoxic Properties

Ozkan et al., (2014) planned to evaluate the genotoxic and antigenotoxic activities of essential oil extracted from the leaves and flowers of *Ferula orientalis* L. grown in Erzurum. The chemical constituents of essential oil were characterized by the GC-MS method. *α*-Cadinol (11.7%), *γ*-cadinene, germacrene D-4-ol, epi-*γ*-muurolol (*α*-pinene 9.3%, 11.9%, 6.1%) were recorded as the main chemical components in leaf (10.45, 8.1, 6.8, 5.9, and 5.7%, respectively) and flower (9.3, 11.9, 6.1, and 7.2%, respectively) essential oils. According to their results, the chemical constituents were responsible for biological activities. Bacterial strains such as *Salmonella typhimurium* TA1537, *S. typhimurium* TA1535, and *E. coli* WP2 uvrA were used to evaluate the mutagenic activity, using the bacterial reverse mutation assay method. The study showed that tested leaf and flower essential oil did not have any mutagenic activity on *S. typhimurium* and *E. coli* strains at any used concentration. Nevertheless, the essential oil showed antimutagenic property against used mutagen, namely N-methyl-N′-nitro-N-nitrosoguanidine (MNNG), 9-aminoacridine (9-AA), and sodium azide (NaN_3_). Further investigations showed the potential of the essential oils to reduce the effect of mutagens on bacterial strains which were as follows: N-methyl-N′-nitro-N-nitrosoguanidine on *E. coli* WP2 uvrA (23–52%); 9-aminoacridine on *S. typhimurium* TA1537 (40–68%), sodium azide on *S. typhimurium* TA1535 (29–36%).

## Scolicidal Activity

In a study, Tabari et al., (2019) revealed the effectiveness of *F. gummosa*'s essential oil and its main components against *Echinococcus granulosus* protoscoleces. Results of GC/MS displayed *β*-pinene as an abundant chemical compound of the essential oil. Furthermore, the eosin staining method was used to measure mortality rate. The mean death rate of *E. granulosus* protoscoleces was recorded 100% at 50 μg/ml concentrations of the essential oil and 60 min exposure time. The essential oil of *F. gummosa* also showed a higher toxic effect on *E. granulosus* protoscoleces with 50% LC_50_ values (lethal concentration) 17.18 μg/ml. Additionally, 10 μg/ml concentrations of only *β*-pinene resulted in the death of tested microorganisms with more than 80% mortality rate. The collective toxic effect of *β*-pinene was efficiently greater than the compressive effect of all chemical compounds presented in the essential oils of *F. gummosa*. On the basis of LC_50._ values (2.20 μg/ml) of *β*-pinene was considered as the most potent scolicidal agent in this study. Kavoosi et al., (2013) demonstrated the scolicidal effectiveness of essential oil from *F. assa-foetida* with a plant. A sulfur-containing hydrocarbon (E)-sec-butyl propenyl disulfide, 62.7%) was found to be the main component in the essential oil analyzed by the gas chromatography method. The results proved that 10 min exposure of *F. assafoetida* essential oil with a concentration of 60 μg/ml or more could kill all *Echinococcus granulosus* protoscolices.

## Toxicity

Ye et al., (2011) studied the effect of chemical components separated from crude essential oil of *F. sinkiangensis* K.M.Shen, on acute toxicity in morphine-dependent animals. Two sulfur-containing compounds: 2-butyl *cis*-1-propenyl disulfide and 2-butyl *trans*-1-propenyl disulfide (SBD) were separated from the unrefined essential oil. To evaluate the effectiveness and naturally abstinent and naloxone-precipitated abstinent morphine-dependent models were applied and injected with 2-butyl *trans*-1-propenyl disulfide intraperitoneally. In addition, the antinociceptive effects, sedative effects, and acute toxicity of SBD were investigated by a writhing test, spontaneous activity test, and LD_50_ values, respectively. The result showed that SBD could be inhibited by the abstinent syndromes, and the 2-butyl *trans*-1-propenyl disulfide had great sedative and antinociceptive effects as well. Tempark et al., (2016) indicated that topical administration of *F. assa-foetida* oleo-gum-resin essential oil could cause contact dermatitis in infants. This high content of disulfide-containing hydrocarbons in asafoetida oil might cause skin-irritating effects. These types of pro-inflammatory side effects could be diminished by the elimination or reduction of disulfide compounds from the essential oil.

## Antioxidant Activity

Kavoosi et al., (2012) demonstrated the radical scavenging properties of *F. assa-foetida*. The essential oil was prepared by hydrodistillation contained (E)-sec-butyl propenyl disulfide and *β*-ocimene and pinene. The essential oil was subjected to different radical scavenging activity assays to evaluate the antioxidant potential. The report displayed that *F. assa-foetida* essential oils did not have any significant antioxidant activity. Pavlović et al., (2012) effectuated antioxidant potential of essential oil obtained from *Ferula heuffelii* Griseb. ex Heuff. underground parts. The main compounds of the essential oil were elemicin and myristicin with 35.4 and 20.6% total concentration. l-Ascorbic acid was used as a reference substance. The resulting investigation delineated that essential oils showed antiradical activity, concentration-dependently. The SC_50_ value was obtained at 22.43 and 3.80 μg/ml for tested essential oil and reference substance, respectively. Ahmadvand et al., (2014) revealed the antioxidant potential and chemical profile of *F. assa-foetida* leaf essential oil. Analysis of hydro-distilled leaves’ essential oil revealed major chemical compounds namely eremophilene (31.28%), *δ*-cadinene, longiborneol, dehydro aromadendrene, and isoledene. Antioxidant activity was examined by using a 0.01–1,000 μg/ml essential oil concentration. Antioxidant activity analysis showed that the IC_50_ for DPPH (2,2-diphenyl-1-picrylhydrazyl) free radicals was 2,375.66 ± 5.13 μg/ml. Benchabane et al., (2012) analyzed the antioxidant potential of *Ferula vesceritensis* Coss. & Durieu ex Trab. The chemical profile displayed viridiflorol (13.4%) as a major constituent of oil and delta-cadinene, *trans*-farnesol, alpha-fenchyl acetate, aristolene, cadinol, and fonenol were also found in the essential oil. Antioxidant activity was accomplished by using a 100–1,000 mg/L oil concentration. Butylated hydroxytoluene (BHT) was used as a positive control with 100–1,000 mg/L concentrations. *F. vesceritensis* essential oil exhibited a reduction in DPPH free radical concentration but with lower efficacy compared to BHT. Znati et al., (2017) assessed different bioactivities of seed oil from *Ferula tunetana* Pomel ex Batt. including antioxidant bioactivity. Pinene (*α*/*β*) and (Z)-*β*-ocimene were characterized as the main component of the essential oil by gas chromatography and carbon-13 nuclear magnetic resonance methods. The essential oil displayed antioxidant activity with moderate efficiency. The H_2_O_2_ assay exhibited the highest activity with 78.2 ± 2.98 μg/ml IC_50_ value, while ABTS, DPPH, and superoxide anion assay radical scavenging assays showed 234.2 ± 12.9, 243.1 ± 6.5 and 89.2 ± 3.82, µg/mL IC_50_ values, sequentially. Amiri (2014) elucidated the antioxidant activity of methanolic extracts and essential oil of *Ferula microcolea* (Boiss.). *α*-Pinene (27.3%), *β*-pinene, nonanaldehyde, *β*-caryophyllene, and 2-isopropyl-5-methylphenol were recognized as primary chemical constituents. The IC_50_ value for *β*-carotene-linoleic acid and DPPH were recorded at 55.2 ± 0.4% and 253.1 ± 2.2 μg*/*ml, for essential oil content, respectively. Kose et al.,(2010) accomplished the antioxidant potential of *Ferula lycia* Boiss. essential oil. The bleaching of the carotene–linoleate and DPPH was used to examine the antioxidant activity of the essential oil. The essential oil (0.4, 1.0 and 2.0 mg/ml) showed *β*-carotene linoleic acid abilities (5.69 ± 2.04, 16.16 ± 0.52, and 27.77 ± 2.37 mg/ml) and DPPH radical scavenging (11.05 ± 0.50, 1.91 ± 0.43 and 2.81 ± 0.0 mg/ml), respectively. Dadaşoğlu et al.,(2018) revealed the antibacterial and antioxidant activity of the essential oil of *F. communis* L. The essential oil showed antioxidant activity at 40.65% (0.1 ml) and 85.16% (0.2 ml) concentration for DPPH, ABTS assay, respectively. Jahani et al.,(2015) synthesized gelatin nano-capsules using *F. assa-foetida* essential oil (FAO) and tested their potential to exhibit antibacterial and antioxidant activity. Essential oils containing gelatin nano-capsules were synthesized with *Ferula* oil at 2, 4, 6, and 8% w/w concentrations; 25% w/w, glutaraldehyde (a cross-linker) and glycerol (plasticizer). Synthesized gelatin nano-capsules were evaluated by scanning electron microscopy. FAO incorporated gelatin nano-capsules exhibited excellent antioxidant and antibacterial at 8% of FAO concentration. Sharopov et al.,(2015) examined the antioxidant activity, along with the anti-inflammatory activity of essential oils of some aromatic plants including *Ferula clematidifolia* Koso-Pol., *Ferula foetida* (Bunge) Regel, etc*.* The results showed that both species had moderate antioxidant activity. Kavoosi and Rowshan (2013) also investigated the chemical profile and antioxidant potential of essential oil from *F. assa-foetida* oleo-gum-resin. *F. assa-foetida* resin (oleo-gum-resins, ORGs) was collected in three different times named as ORG1, ORG2, and OGR3 and subjected to hydro-distillation. The IC_50_ value for all listed scavenging methods were calculated as follows OGR1 (0.017 ± 0.0019, 0.012 ± 0.0020, 0.035 ± 0.0027, and 0.022 ± 0.0012 mg/ml); OGR2 (0.031 ± 0.0018, 0.025 ± 0.0023, 0.047 ± 0.0028, and 0.033 ± 0.0043 mg/ml); OGR3 (0.047 ± 0.0028, 0.035 ± 0.0012, 0.066 ± 0.0042, and 0.055 ± 0.0038 mg/ml) for RNS, ROS, TBARS, and H_2_O_2_ scavenging assay, respectively. Antioxidant activity of OGR1 (18.16 ± 1.2 mg), OGR2 (14.14 ± 2.2 mg), and OGR3 (10.8 ± 2.5 mg) were observed at mg ascorbic acid/g of essential oil, respectively. Topdas et al.,(2020) revealed the antioxidant activity of *Ferula orientalis* L. *essential oil* containing *α*-pinene, *ortho*-cymene, limonene, terpinolene, *β*-caryophyllene and isobornyl acetate as the major compounds*.* The antioxidant activity (*in vitro*) of the essential oil was analyzed. The report averred that the essential oil chemical group had significant antioxidant activity against the ABTS and DPPH free radicals. Sharopov et al.,(2019) revealed the antioxidant activities of hydrodistilled essential oil, extracted from the *Ferula tadshikorum* Pimenov. underground parts. Data analysis confirmed that the essential oil exerted lower antioxidant potential than the caffeic acid (positive control) with 17.8 and 8.2 mg/ml median inhibitory concentration (IC_50_) for DPPH and ABTS, respectively.

## Antiprotozoal Activity

Essid et al.,(2015) investigated the antileishmanial activity of essential oil from medicinal plants. This study included *Ferula communis* L. with 11 medicinal plants to identify the antileishmanial potential against *Leishmania infantum* and *L. major* promastigotes. The major components of *F. communis* were: *β*-caryophyllene (15.22%), *β*-myrcene alpha-eudesmol, alpha-pinene, *para*-mentha-1, 5-dien-8-ol and γ-curcumene. Amphotericin B was applied as a form of positive control in the experimental setup. Data collected from the experiment setup showed that the essential oils had potent antileishmanial activity against *L. infantum* and *L. major* promastigotes at IC_50_ value <1 μg/ml. After result analysis, it was concluded that *L. infantum* promastigotes (IC_50_ value 0.80 ± 0.18 μg/ml) were more sensitive to the essential oil and their constituents as compared to *L. major* (IC_50_ value 0.22 ± 0.09 μg/ml). According to Essid et al., caryophyllene had high leishmanicidal activity against *L. infantum* and *L. major* (1.06 ± 0.37 and 1.33 ± 0.52 μg/ml, respectively). Andrade et al.,(2016) effectuated the *in vitro* anti-leishmanial activity of different plants’ essential oils including *Ferula galbaniflua* Boiss. & Buhse. GC–MS analysis of the essential oil showed methyl pimar-8-en-18-oate (41.82%) and diethyl phthalate (13.09%) as main components. *In vitro* leishmaniacidal activity of essential oil was examined on *Leishmania amazonensis* promastigotes forms at 30–500 μg/ml oil concentrations. *F. galbaniflua* essential oil was more potent against *L. amazonensis*at at 95.70 ± 1.82 μg/ml (IC_50_/24 h). El-Deeb et al.,(2012) substantiated *in vitro* inhibitory effects of *F. assa-foetida* essential oil on *Blastocystis* species. The volatile oil of powdered assa-foetida was extracted by hydrodistillation and tested against the *Blastocystis* sp. subtype. Various concentrations such as 5, 10, 25, 40, and 50 mg/ml were used for 24, 72, and 144 h. Metronidazole was used as the reference antiprotozoan drug including a 10, 100, and 500 μg/ml concentration. The results confirmed by microscopy described that extracted oil decreased the viability and counts of all the *Blastocystis* sp. The lowest and highest percentage inhibition values at which blastocysts showed no growth and multiplication were 16 and 40 mg/ml, respectively. At the aforementioned concentration, the mean count was the same for the oil extract and the reference drug. Furthermore, re-cultivation of *Blastocystis* in oil-free medium did not display any growth even after 48, 72, and 144 h of cultivation.

## Neuroprotective

Topdas et al.,(2020) elucidated the neuroprotective potential of various types of extracts and essential oils of *F. orientalis* L. Neuroprotective potential of the essential oil was investigated in cortex neuron cells by 2-(3,5-diphenyltetrazol-2-ium-2-yl)-4,5-dimethyl-1,3-thiazole; bromide (MTT) assay. The essential oil concentrations ranging from 10^−1^ to 10^−8^ were used for the experiments. The cell groups treated with essential oil clearly exhibited the highest cell viability rates. The viability rates were 92.57 ± 4.23, 91.29 ± 4.12, and 83.60 ± 3.98% at 10^−2^, 10^−3^, and 10^−4^ of the oil concentration (*p* > 0.05), respectively. Moreover, the result clearly showed that at 10^−2^ concentration, the cell viability was at its peak, after this mentioned concentration of essential oil, the viability rates started to fall down slightly.

## Antigerminative Activity

The *in vitro* antigerminative property of *Ferula tunetana* Pomel ex Batt. seed oil was demonstrated by Znati et al.,(2017). Four doses (1.25, 5, 10, and 20 mg) of the oil were prepared by diluting it in the emulsion in 5 ml deionized H_2_O. The essential oil exerted significant toxicity against *Medicago sativa* L., *Triticum aestivum* L. and *Lactuca sativa* Linn*.* Seven days of exposure to essential oil showed the maximum toxic effect with 0% germination. Furthermore, the germination of *M. sativa*, *T. aestivum* and *L. sativa*, seedlings were efficiently inhibited at a 20 and 10 (mg/5 ml) concentration. The authors suggested that *α*-pinene (39.8%) was responsible for the toxic effect.

## Vasodilatory or Vasorelaxant Activity

Esmaeili et al.,(2020) studied the role and significance of the K^+^channels in vasorelaxant and the effect of essential oil from asafoetida (AEO). The AEO obtained from *F. assa-foetida* oleo-resin was subject to the vasodilation effect examination. This effect had two types (endothelium-independent and dependent). This research was designed to demonstrate whether intracellular Ca^2+^ release and K^+^ channels had a contribution in the essential oil-mediated vasodilation or not. For the experiments, isolated rats' thoracic aortas were denuded, and the concentration-response curve was plotted after induction of contraction by KCl (60 mM) and addition of 0.625–80 μL/L of AEO in the medium. The vasodilatory effect of the essential oil was studied by the addition of essential oil in the medium before and after the addition of phenylephrine and potassium channel blocking chemicals viz. barium chloride (BaCl_2_), glibenclamide (GL) and 4-aminopyridine (4A). AEO displayed a relaxant effect on the precontracted rings, concentration-dependently at a 23 μL/L IC_50_ value. Furthermore, it was observed that K^+^ channel blockers significantly abolished the AEO mediated vasodilatory effect, if it was added before the addition of KCl to the ring medium. In contrast to potassium channel blockers, the tension was significantly decreased with the addition of the AEO before or after phenylephrine addition. The result interpreted that the inhibition of Ca^+2^ channels and the activation of smooth muscle membrane K^+^ channels were responsible for the vasodilatory effect of the essential oil on the denude *γ*-endothelium aortic ring. Esmaeili et al.,(2017) investigated the vasodilatory activity of AEO using rat aorta ring as a living system. The contribution of Ca^+2^ channel, prostacyclin and NO (nitric oxide) in the vasodilation process were additional. In an experiment, thoracic aorta rings were stretched in an organ bath apparatus, after that the rings precontracted using 80 mM of KCl with or without the AEO portion. To reveal the role of nitric oxide and prostacyclin in the AEO vasodilatory effect, indomethacin (blocker of cyclooxygenase) and l-NAME (NO synthase blocker) were used. The AEO effect on the influx of Ca^+2^ ions were also evaluated. Data showed that the essential oil exerted significant effects on the aorta rings’ vasodilation; the IC_50_ values for denuded and intact endothelium cells were 19.2 and 1.6 μL/L, sequentially. The AEO mediated vasodilatory could reduce by l-NAME or indomethacin but could not be abolished. On the basis of the result, it was concluded that the AEO had an effective vasodilation activity, which could be endothelium-dependent or independent. AEO also decreased the influx of Ca^+2^ ions from the calcium channels of the plasma membrane into the cell.

## Antiepileptic or Anticonvulsant and Hypotensive Effect

Sayah et al.,(2001) used the fruit essential oil of the *F. gummosa* to evaluate antiepileptic activity. The result showed that the essential oil had no measurable effect on maximal electroshock-induced seizures, but it can reduce the effect of pentylenetetrazole-induced tonic seizures in mice. GC analyses of the essential oil presented pinene (*β*: 50.1%; *α*: 18.3%), delta-3-carene (6.7%), origanene (3.3%), and 4(10)-thujene (3.1%) as the main components. It was also suggested that toxic and anticonvulsant effects of the essential oil might be related to the compounds *α*-thujene and *β*-pinene. Ghanbari et al.,(2012) aimed to reveal the chronic and acute effects of *Ferula persica* Willd. on hypertensive rats’ blood pressure. Their study presented that the hypotensive effect might be due to the presence of safranal, a component of the *F. persica* EO. Furthermore, it was found that *F. persica*’s essential oil might exert a hypotensive effect by the induction of nitrous oxide release and muscarinic receptors targeting in rats.

## Hypoglycemic and Hypolipidemic Effect

Heydari-Majd et al.,(2019) performed the synthesis of *F. gummosa* essential oil or barije essential oil (BEO) incorporated zein nanofibre, subjected to *α*-amylase and *α*-glucosidase inhibitory action. GC/MS analysis of BEO revealed the presence of alpha-pinene, guai-1 (10)-en-11-ol, champacol and *β*-myrcene as the major components of the oil. Morphological analysis of prepared zein nanofibers done by SEM and FT-IR showed that the essential oil components were successfully entangled in the ribbon structured zein fibers with ∼95% encapsulation efficiency. BEO-loaded (1–4% w/w) zein nano-fibers exhibited *α*-amylase (IC_50_: 1.09 ± 0.02 to 1.64 ± 0.01 mg/ml) and *α*-glucosidase (IC_50_:0.78 ± 0.01 to 1.25 ± 0.03 mg/ml) inhibition activity. The model-fitting results showed that BEO-loaded zein nano-fibers could be a delivery vehicle for diabetes control. Karimlar et al.,(2019) exposed the hypoglycemic and hypolipidemic effects of three medicinal plants' essential oils including *F. gummosa* on streptozotocin-induced diabetic rats. For the experimental setup the streptozotocin-induced (45 mg/kg doses, intraperitoneally) male wistar rats were used and treated with the essential oil (200 mg/kg/day). After 30 days, rats' lipid profiles and serum glucose were assessed. Data were examined by the Tukey test and one-way ANOVA test. The value of HDL-C, LDL-C, cholesterol, triglyceride, and glucose in *F. gummosa* essential oil treated rats’ blood were recorded as 42.07 ± 9.68, 29.59 ± 3.76, 91.02 ± 11.95, 105.18 ± 12.13, and 429.91 ± 46.14 µM, respectively. The *F. gummosa* essential oil significantly reduced low-density lipoprotein cholesterol and triglycerides in diabetic rats; even though the essential oil tested group did not display any significant difference in glucose level from the diabetic group. Yarizade et al.,(2017) studied the antidiabetic activity of *F. assa-foetida*. In this study, it was observed that *F. assa-fetida* showed its antidiabetic activity by inhibiting *a*-glucosidase and DPP-IV (Dipeptidyl peptidase- IV).

## Antinociceptive and Hyperalgesia Effect

In a study, Radulović et al.,(2013) reported hyperalgesia induction in mice with a chemical compound isolated from *F. ovina* (Boiss.) Boiss. The chemical profile of aerial parts of the essential oil of the *F. ovina* revealed the presence of a rare aromatic ester of monoterpenic alcohol named bornyl 4-methoxybenzoate, and its structure was evaluated by X-ray crystallographic analysis. The analgesic effect (the hot plate and tail immersion tests) and antinociceptive activity (abdominal writhings test) of the new compound with other oil constituents were elucidated. To determine the effect of essential oil and bornyl 4-methoxybenzoate, an experiment was performed using male BALB/c mice as laboratory models. The results showed that bornyl 4-methoxybenzoate induced hyperalgesia in mice which is revealed by a hot plate test. The transient receptor channels (TrpV3) could have a target for tested substances and is a possible reason for hyperalgesia. The oil was found to have exerted a modest central and significant peripheral analgesic effect. The oil rendered a significant antinociceptive activity, dose-dependently, and abolished acetic acid-induced abdominal writhings. The number of writhings was reduced by up to 92% at 200 mg/kg essential oil concentration and up to 83% at 200 mg/kg bornyl 4-methoxybenzoate concentration in treated mice was observed. Some of the major chemical constituents such as myrcene, limonene, and *α*-pinene were ascribed to possess certain analgesic potential. The result showed that essential oil and bornyl 4-methoxybenzoate could have caused inhibition of prostaglandin synthesis. Bagheri et al.,(2016) studied the antinociceptive potential of *F. assa-foetida* seed essential oil in mice. To evaluate the antinociceptive effect of the essential oil (2.5, 5 and 10 mg/kg), acetic acid-induced writhing and a hot plate test were used and for the control group morphine sulfate (8 mg/kg) or sodium diclofenac (30 mg/kg) were applied. Hot plate testing results showed that the percentage of the MPE (maximum possible effect) was higher for all used concentrations of the essential oil than morphine sulfate and sodium diclofenac. The writhes numbers were significantly less in the essential oil treated mice as compared to the control group. This research findings indicated that the essential oil reduces acetic acid-induced writhes numbers dose-dependently and presented a potent antinociceptive effect on acute/chronic pain in mice. The analgesic effect of the essential oil is thought to be either due to its action on acetic acid-sensitive visceral receptors or inhibition of synthesis and action of prostaglandins and also cyclooxygenase and/or lipoxygenase in the arachidonic acid cascade at the peripheral route.

## Anticancer and Antitumor Activity

Hosseinzadeh et al.,(2020a), Hosseinzadeh et al.,(2020b) performed the synthesis of gold nanoparticles using essential oil obtained from the gum of *F. persica* Willd. and evaluated their *in vitro* anticancer effects. Phytochemistry profiles were effectuated by the GC- MS method and displayed 27 constituents such as *α*-pinene (27.1%), (Z)-sec-butyl propenyl disulfide (20.2%) and *β*-pinene (10.6%) as the major component. The gold nanoparticles (Au NPs), which were characterized by ultraviolet-visible spectroscopy showed absorption at 530 nm. The shape (spherical) and size (37.05–78.6 nm) of Au NPs were confirmed by TEM image. The presence of reducing and capping essential oil compounds on the gold ions and metal crystal structure was revealed by the FTIR spectrum and XRD pattern, respectively. The apoptosis and cytotoxicity assessment were performed by MTT [3-(4,5-dimethylthiazol-2-yl)-2,5-diphenyl tetrazolium bromide] assay and AO/EB (acridine orange/ethidium bromide) staining using non-cancerous (Vero cells) cells and cancerous (Murine colon carcinoma CT26) cells. The result showed that the cytotoxicity effect of Au NPs was dose-dependent and exhibited cytotoxicity against Vero cell and murine colon carcinoma CT26 lines with IC_50_ values 0.0024 and 0.0307 mg/ml, respectively. Further, AuNPs inhibited colony formation in the aforementioned cells and induced apoptosis. The effect of AuNPs was reported more intensively against CT26 cells. The result clearly indicated the cytotoxic, apoptotic, and antiproliferative potential of the synthesized Au NPs. Elghwaji et al.,(2017) aimed to study the antitumor potentiality of the *Ferula tingitana* L. of essential oil. The essential oils were obtained from the hydrodistillation of flower and leaves possessing sesquiterpenes and oxygenated sesquiterpenes as their major chemical components, respectively. Cytotoxicity against human tumor cells viz. hormone-responsive breast (MCF7) cells, cervical (HeLa) cells and liver carcinoma (HePG2) cells were performed by sulforhodamine B (SRB) method using different doses of the essential oil (0.0–50.0 μg/ml). The IC_50_% value for *in vitro* cytotoxicity of flower and leaf derived essential oils against liver carcinoma (HEPG2), cervical (HELA), and breast (MCF7) cell lines (two different) were 4.4, 4.2; 8.6, 10.9 and 6.9, 4.8 μg/ml, respectively. Dithiolane found in high concentrations in *F. assa-foetida* essential oil; this compound exhibited antiproliferative activity in two human liver carcinoma cell lines (SK-Hep1and HepG2), dose-dependently. Two signaling molecules: NF-kB and TGF-β altered after the use of bioactive compounds of *F. assa-foetida*; moreover, an increase in caspase-3 and TNF-α expression was observed and caused induction of apoptosis (Verma et al., 2019).

## Cytotoxic Activity

The chemical profile and bioactivity of essential oil from underground parts of the *Ferula tadshikorum* Pimenov. were investigated (Sharopov et al., 2019). The chemical profile analysis revealed sulfur-containing hydrocarbon as a major component. The assessment of the cytotoxic effect of the essential oil was done on CEM/ADR5000 and CCRF-CEM cancer cell lines by using an MTT assay. Data displayed IC_50_ values were 142.5 μg/ml and 21.6 μg/ml for CEM/ADR5000 and CCRF-CEM cell lines, respectively. The essential oil shows a reduced cytotoxicity effect on CEM/ADR5000, due to the presence of substrates P-glycoprotein (p-gp) and over-expression of ATP-binding cassette transporter p-gp, and it rapidly pumped all the active molecules of essential oil out of the cells. Ben et al.,(2016) elucidated the cytotoxic effect of the root oil obtained from the *Ferula lutea* (Poir.) Maire. The chemical profile of the oil was investigated by GC-MS/FID and 13C-NMR spectroscopy revealed the major component viz. delta-3-carene (**∼**73%). The cytotoxic effect of the essential oil was carried out using the MTT method on human colon cell lines (HCT-116 and HT-29 cells). For the testing, Paclitaxel was added as a positive control. The result of MTT assay delineated that the hydrodistilled *F. lutea* roots’ essential oil has a moderate cytotoxic effect on HT-29 and HCT-116 cells (Khajeh et al., 2005). The IC_50_ values were 26.39 ± 3.98 μg/ml and 81.00 ± 12.81 μg/ml for HT-29 and HCT-116, respectively. The result showed that HCT-116 cells were extra sensitive to the isolated essential oil compared to HT-29 cells. The cytotoxicity of gum essential oil from *F. persica* Willd. on Vero cell lines and murine colon carcinoma (CT26) was demonstrated by MTT assay (Hosseinzadeh et al., 2020a; Hosseinzadeh et al., 2020b). The primary component of the essential oil was alpha-pinene (27%), (Z)-sec-butyl propenyl disulfide (20%), *β*-pinene (11%), *trans-*dihydroagarofuran (6%), allo-aromadendrene (5%), (Z)-*β*-ocimene (4.5%) and *α*-caryophyllene (3%). The viability was decreased in the Vero cell (0.125 × 10^−9^to 80 μL/ml) and CT26 cell line (0.125 × 10^−9^ to 20 μL/ml) significantly with all different concentrations of the essential oil treatment, dose-dependently. The IC_50_ value for the CT26 cell line (0.3247 μL/ml) was relatively greater than Vero cells (0.0010 μL/ml). The essential oil treatment exhibited valuable inhibition of colony formation in Vero cells and CT26 compared to the control. Moreover, some measurable changes such as morphological changes, the monolayer of the cells with areas devoid of cells, nucleus condensation, blebbing of the cell membrane, and apoptotic body formation were observed in fluorescence microscope by AO/EB staining. Bagheri et al.,(2017a) studied the cytotoxic effect of ferulic acid and essential oil obtained from oleo-gum resin of *F. assa-foetida* on 4T1 breast cancer cells. Data analysis enlightened the fact that the incubation of breast cancer 4T1 cells with the essential oil at the concentration ranging from 1 to 1,000 μg/ml for 24 h did not show any significant cytotoxicity. Additionally, the viability of cancer cells started to gradually decrease after 48 and 72 h of the incubation period. Nevertheless, a 10% cell viability rate was remarked even after the incubation with the highest concentration (1,000 μg/ml) of the essential oil for 72 h. On the basis of the result, it could be concluded that the cytotoxic effect of the essential oil was time and concentration-dependent. Nguir et al.,(2016) used the Hela cervix cell and A549 human lung epithelial carcinoma cell lines to assess the cytotoxicity of *F. communis* L. essential oil (flowers, roots, leaves, and stems). The MTT test method was used with slight modifications.

For the activity assessment, the cell lines were treated with the essential oil at different concentrations. It was observed that activity was increased in both cell lines with a higher essential oil concentration, but at 500 μg/ml concentration significant activities were observed in both cell lines. Moreover, the Hela cells were reported to be more sensitive than A549 cells and displayed 79.05% and 77.52% inhibition at 500 and 250 μg/ml concentration of stem essential oil, respectively; flower essential oil showed 74.89% inhibition. Abbaszadegan et al.,(2015) demonstrated the cytotoxicity of *Ferula gummosa* essential oil against L929 mouse fibroblasts using a colorimetric, MTT assay, and the Sigma-Aldrich method. Chlorhexidine (CHX: 0.2%) and sodium hypochlorite (NaOCl: 5%) solutions were used as a control. The cytocompatibility of the essential oil was estimated on L929 fibroblast cells in comparison to the control. The chemical profile analysis of the essential oil displayed the presence of 27 chemical constituents. *β*-Pinene (51.83%) was the main component in the essential oil. In the experiment, culture medium and H_2_O_2_ (35%) were used as the negative and positive controls, respectively. The cytotoxicity assessment showed that the full concentration (50 μL/ml) of the essential oil had the ability to keep the mean cell viability of L929 mouse fibroblast cells at about 88%. There was no measurable difference in the mean viability, of the negative control group (H_2_O_2_) and in NaOCl (5%) and CHX (0.2%) treated cells. Additionally, no significant variation between the cytotoxic effect of the essential oil and CHX or NaOCl was observed. Kavoosi and Purfard (2013) considered phenolic monoterpenes as a target site for essential oils to show their cytotoxic effect which was the cytoplasmic and mitochondrial membrane. The oil molecules pass through the cytoplasmic membrane and increase permeabilization, and ions leakage (especially, potassium and calcium) from membranes reduces membrane electric potential, ATP, amino acids, proteins synthesis, and cell death.

## Miscellaneous Activity

Rashidi et al.,(2014) demonstrated the galbanum prophylactic effect on caffeine teratogenic effects. For the experiment, four groups of pregnant rats were selected: one group (control) was injected with saline, two groups with galbanum (200 mg/kg), caffeine (80 mg/kg), and the remaining group with both compounds (galbanum + caffeine), intraperitoneally for 9–11 days of gestation. To obtain the data, fetuses (20th day of gestation) were collected and stained using Alizarin red-Alcian blue method. The report established that galbanum decreased caffeine-induced cleft palate incidence by 8.3% in the galbanum group as compared to the caffeine group (33.3%). Kavoosi et al.,(2013) tried to develop *F. assa-foetida* essential oil incorporated wound dressing film with potent antibacterial and antioxidant properties. The film was prepared with 10% w/v gelatin solutions containing different concentrations of the essential oil. The result analysis displayed that the entraption of essential oil into gelatin films showed a valuable reduction in tensile strength swelling, elastic modulus and vapor penetrability; enhancement in solubility and resistance. However, essential oil incorporated gelatin film displayed appreciable antioxidant and antimicrobial activities compared to gelatin film without essential oil. In a study, Esmaeili et al.,(2018) investigated the effect of the essential oil on a myocardial ischemic-reperfusion injury obtained from *F. assa-foetida* (AEO). Three concentrations of AEO (0.50, 0.25 and 0.125 μL/g heart) were used. The results of the analysis showed that the AEO treated group exhibited severe myocardial dysfunction with a significant increase in left ventricular end-diastolic pressure and a reduction in left ventricular developed pressure as compared to the control group. The markers of myocardial injury (lactate dehydrogenase and creatine kinase) were also significantly active in the treated group compared to the control. Moreover, the essential oil exerted an effect on perfusion in isolated rat hearts at 0.5 μL/g heart concentration, but not below these concentrations.

## Conclusion and Future Perspectives

The trend of natural products in the medical and other fields is growing and replacing the use of chemically synthesized drugs. Therefore, numerous scientific investigations have been conducted over a few decades. On the basis of the literature, the genus *Ferula* is a well-known genus of the Apiaceae family. It is widely used as an aroma spice in different foods all over the world. To date, a large number of biologically active components have been separated from the essential oils of *Ferula* which have shown the many different activities such as antimicrobial, insecticidal, antioxidant, antigerminative, cytotoxic, antitumor, antidiabetic activities, etc. All these activities have been discussed in this review. A large number of reports have been conducted on the variability in the chemical composition (quality and quantity) of essential oil extracted from the members of the genus *Ferula*. In these reports, it has clearly been stated that environmental factors, genetic factors, geographical area, and collection time have a great effect on essential oil composition. *Ferula* essential oil showed great anti-cancerous activity against various tested cancer cell lines. After a literature survey on the antimicrobial and insecticidal potential of this genus, it can be concluded that the essential oils are effective drug candidates for developing natural or semi-synthetic derivatives against drug-resistant microbes and insects and protozoan (*Leishmania*). Along with all the positive points, there are some negative points associated with essential oils derived from this genus which should be considered. Some species of *Ferula* show toxicity in humans and animals. Further, research is therefore needed in order to develop a befitting standard of the safe use of essential oils. The pharmaceutical potential of the essential oil of the *Ferula* species is still not completely understood. The revealed bioactivities are reported *in vitro*, therefore their practical application should be focused on and detailed studies are also needed to find new chemical constituents in the genus.
